# Distinct Long- and Short-Term Adaptive Mechanisms in Pseudomonas aeruginosa

**DOI:** 10.1128/spectrum.03043-22

**Published:** 2022-11-14

**Authors:** Michal Koska, Adrian Kordes, Jelena Erdmann, Sven D. Willger, Janne G. Thöming, Heike Bähre, Susanne Häussler

**Affiliations:** a Department of Molecular Bacteriology, TWINCORE, Centre for Experimental and Clinical Infection Research, Hannover, Germany; b Department of Molecular Bacteriology, Helmholtz Centre for Infection Research, Braunschweig, Germany; c Department of Clinical Microbiology, Copenhagen University Hospital—Rigshospitalet, Copenhagen, Denmark; d Research Core Unit Metabolomics and Institute of Pharmacology, Hannover Medical School, Hannover, Germany; e Cluster of Excellence RESIST (EXC 2155), Hannover Medical School, Hannover, Germany; Griffith University

**Keywords:** *Pseudomonas aeruginosa*, small-colony variant, alginate, biofilms, chronic infection, cyclic di-GMP, hysteretic memory response

## Abstract

Heterogeneous environments such as the chronically infected cystic fibrosis lung drive the diversification of Pseudomonas aeruginosa populations into, e.g., mucoid, alginate-overproducing isolates or small-colony variants (SCVs). In this study, we performed extensive genome and transcriptome profiling on a clinical SCV isolate that exhibited high cyclic diguanylate (c-di-GMP) levels and a mucoid phenotype. We observed a delayed, stepwise decrease of the high levels of c-di-GMP as well as alginate gene expression upon passaging the SCV under noninducing, rich medium growth conditions over 7 days. Upon prolonged passaging, this lagging reduction of the high c-di-GMP levels under noninducing planktonic conditions (reminiscent of a hysteretic response) was followed by a phenotypic switch to a large-colony morphology, which could be linked to mutations in the Gac/Rsm signaling pathway. Complementation of the Gac/Rsm signaling-negative large-colony variants with a functional GacSA system restored the SCV colony morphotype but was not able to restore the high c-di-GMP levels of the SCV. Our data thus suggest that expression of the SCV colony morphotype and modulation of c-di-GMP levels are genetically separable and follow different evolutionary paths. The delayed switching of c-di-GMP levels in response to fluctuating environmental conditions might provide a unique opportunity to include a time dimension to close the gap between short-term phenotypic and long-term genetic adaptation to biofilm-associated growth conditions.

**IMPORTANCE** Extreme environments, such as those encountered during an infection process in the human host, make effective bacterial adaptation inevitable. While bacteria adapt individually by activating stress responses, long-term adaptation of bacterial communities to challenging conditions can be achieved via genetic fixation of favorable traits. In this study, we describe a two-pronged bacterial stress resistance strategy in the opportunistic pathogen Pseudomonas aeruginosa. We show that the production of adjusted elevated c-di-GMP levels, which drive protected biofilm-associated phenotypes *in vivo*, resembles a stable hysteretic response which prevents unwanted frequent switching. Cellular hysteresis might provide a link between individual adaptability and evolutionary adaptation to ensure the evolutionary persistence of host-adapted stress response strategies.

## INTRODUCTION

The ubiquitous bacterium Pseudomonas aeruginosa has emerged as a model organism to study phenotypic and genotypic adaptation to diverse and challenging environmental conditions. P. aeruginosa is a problematic opportunistic pathogen (1, 2). In cystic fibrosis (CF) patients, chronic P. aeruginosa infections contribute to high morbidity and mortality rates (3). Despite even intensified antimicrobial therapy, eradication of the infection is difficult (4). Although most CF patients are initially infected with only one P. aeruginosa clone, selection of beneficial mutations in various niches of the heterogeneous habitat of the chronically infected CF lung causes diversification (5). It is a well-described phenomenon that bacterial P. aeruginosa isolates from CF patients display a wide range of different colony morphologies, among them mucoid and small-colony variants (SCVs) (6). Diversification facilitates adaptation and thus survival of niche specialists at various sites of the infection (7–9). It also guarantees survival of bacterial populations in unpredictable and unstable environments and increases the fitness and general stress response of populations (5, 10, 11).

The mucoid P. aeruginosa phenotype is caused by overproduction of alginate. Alginate, a polymer consisting of l‐guluronic and d‐mannuronic acid residues, protects bacteria from host defenses and phagocytosis, thus contributing to persistence and tolerance within the CF lung (12). Alginate production can be induced as a response to environmental stresses leading to proteolytic degradation of the anti-sigma factor MucA, whereas inactivating mutations in *mucA* fix the mucoid phenotype also under noninducing conditions (6, 13). In addition to mucoid isolates, SCVs are frequently isolated from the chronically infected CF respiratory tract and have been associated with persistent infections (14, 15). Many of the SCVs express further phenotypic features such as hyperpiliation, increased biofilm formation, and increased resistance toward antibiotics, while they exhibit a reduced expression of virulence determinants (16–18).

SCVs of a variety of other bacterial species, such as Staphylococcus aureus, Escherichia coli, Burkholderia pseudomallei, Burkholderia cepacia, Vibrio cholerae, and Neisseria gonorrhoeae, have been recovered from clinical samples, e.g., from the respiratory tract, blood, and soft tissues (reviewed in reference 19). In recent years, many efforts to gain new insights into the molecular mechanisms underlying the switch to a small-colony variant phenotype have been made (20–24). Various studies have demonstrated that the SCV phenotype is often, but not exclusively, associated with elevated intracellular levels of the second messenger bis-(3′-5′)-cyclic dimeric GMP (c-di-GMP) (14, 23, 25–28). c-di-GMP is one of the main drivers in the regulation of the transition from a planktonic, virulent to a sessile biofilm-associated lifestyle (29, 30). In general, high intracellular levels of c-di-GMP activate the production of exopolysaccharides (EPS) and promote adhesion and biofilm formation, whereas low levels of c-di-GMP are associated with high motility, virulence, and a planktonic mode of growth (31).

In this study, we demonstrate a delayed switching of high c-di-GMP levels as well as the expression of alginate biosynthesis in a clinical mucoid SCV isolate, which is reminiscent of a hysteretic response. We discuss a model of a combinatorial influence of genetically fixed mutations and cellular hysteretic c-di-GMP responses on the evolution of mucoid P. aeruginosa SCV phenotypes that are selected *in vivo* under human host infection conditions.

## RESULTS

### Whole-genome sequence comparison between the clinical isolate SCV24 and large-colony-producing revertants.

**FIG 1:**
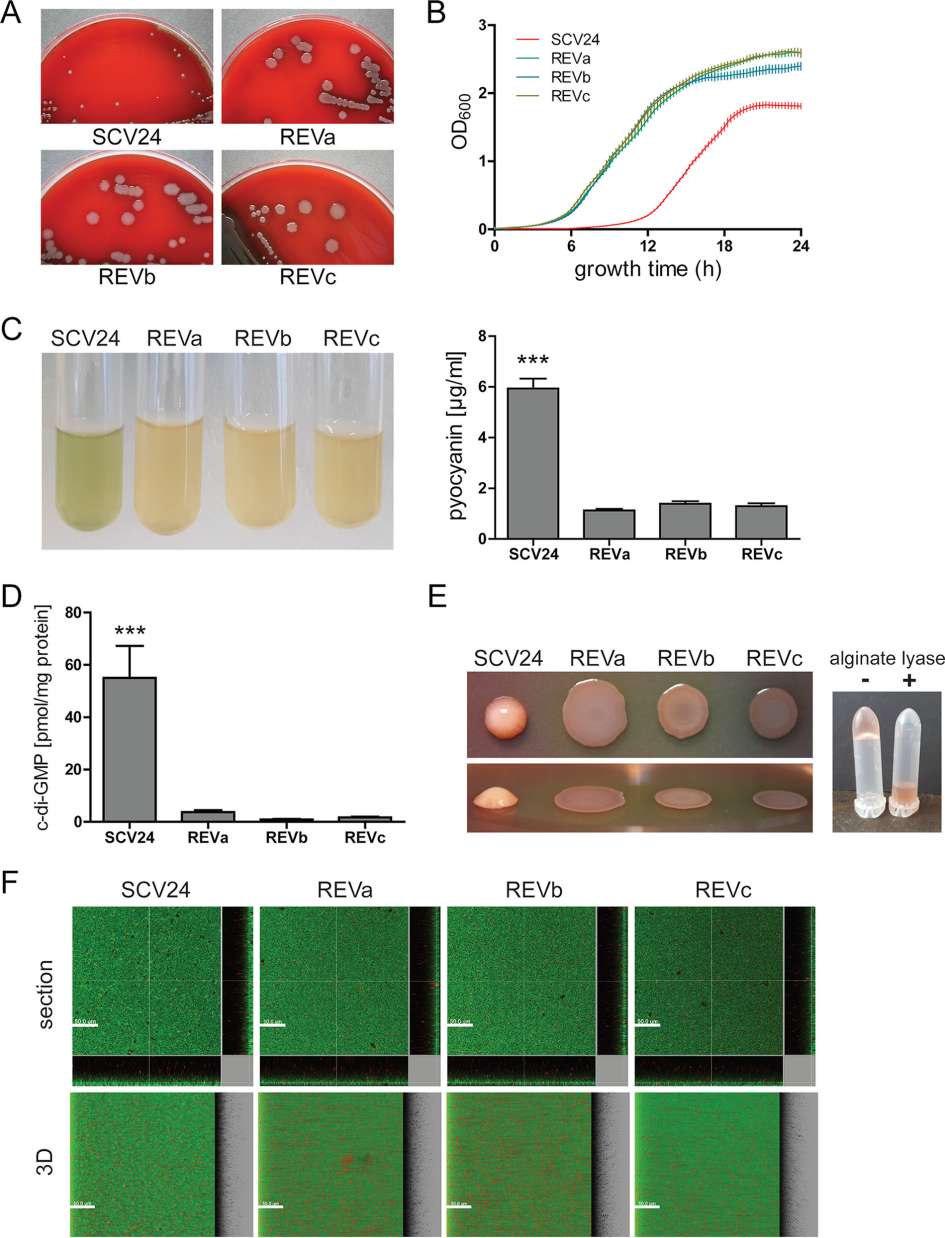
Phenotypic characterization of SCV24 and its revertants. (A) Representative photographs of colonies of the small-colony variant SCV24 and its revertants (REVa to -c). Pictures of the bacterial colonies were taken after 40 h of incubation on Columbia agar plates at 37°C. (B) Growth curves of SCV24 and its revertants cultivated at 37°C in LB were recorded by the use of a Bioscreen device. Average OD_600_ values of 8 biological replicates are shown. Error bars represent the standard deviation. (C) Qualitative and quantitative pyocyanin production in planktonic cultures of SCV24 and its revertants in LB after 24 h is depicted. (D) Intracellular c-di-GMP concentrations were quantified by liquid chromatography-mass spectrometry after 24 h of growth in LB at 37°C. Data represent the mean values from three biological replicates; error bars represent the standard deviations. Levels of statistical significance were calculated using a two-tailed unpaired *t* test (***, *P* < 0.001). (E) Colony morphologies on Congo red agar (0.5%) after incubation for 14 days at room temperature (left). The mucoid matrix of SCV24 was dissolved in PBS and treated with (+) and without (−) alginate lyase (right); the picture of the inverted tubes was taken after 60 s of incubation. (F) Representative microscopic images of 48-h-old biofilms of SCV24 and its revertants grown in 96-well plates. The biofilms were stained with the *Bac*Light bacterial viability kit, visualizing dead cells in red (propidium iodide) and living cells in green (Syto9).

Most clinical P. aeruginosa SCV phenotypes are unstable and revert to large-colony morphologies upon serial passages in rich medium. Here, by subculturing the clinical isolate SCV24 *in vitro*, three independently generated revertants were isolated (Fig. 1A) and subjected to whole-genome sequencing. When comparing the genomic sequence of the revertants to that of SCV24, all three revertants showed exclusively single nucleotide polymorphisms (SNPs) in the *gacA* (PA14_30650) or the *gacS* (PA14_52260, annotated as *lemA* in the PA14 reference genome) gene. Both genes form an operon encoding a two-component system, previously described to be involved in the regulation of the switch between the acute and chronic phases of infection (32). One revertant, REVa, harbored a C-to-T transition 391 bp downstream of the *gacA* start codon, while REVb contained a C-to-A exchange at position 1787 of the *gacS* gene. Both mutations are nonsense mutations, resulting in the formation of a STOP codon (see Table S1 in the supplemental material). REVc exhibited a frameshift mutation within the *gacS* gene due to an insertion of 5 nucleotides (GAAGG) at position 629.

To confirm that the molecular basis for the evolution of revertants in SCV24 is mainly due to inactivating mutations in either *gacA* or *gacS*, we PCR amplified and sequenced the *gacSA* genomic region in 18 additional independently generated revertants. Three revertants harbored sequence variations in *gacA*, 10 in *gacS*, and one (REVs) in both *gacA* and *gacS* (Table S1). Thus, out of the overall 21 revertants which were analyzed in this study, 17 harbored sequence variations within the *gacSA* operon.

### Phenotypic characterization of SCV24 and its revertants.

The clinical isolate SCV24 produces small colonies on agar plates. In addition, it exhibits an extended lag phase in liquid cultures when grown in LB medium and did not reach the same maximum values of optical density at 600 nm (OD_600_) as its revertants (Fig. 1B). Furthermore, whereas SCV24 produced pyocyanin in stationary growth phase, this phenotype was abolished in all revertants (Fig. 1C). The generation of small colonies on agar plates, as well as the production of pyocyanin, has been previously shown to be linked to high levels of intracellular c-di-GMP (33, 34). In accordance with these findings, we observed a significantly lower concentration of c-di-GMP in all three revertants (Fig. 1D).

c-di-GMP also positively regulates the production of exopolysaccharides (EPS), an important extracellular matrix component of biofilms (31). In line with this, SCV24 produced mucoid colonies on agar plates, which became more apparent upon extended incubation time at room temperature. Alginate seemed to be the main component of the mucoid matrix as it could be disrupted and liquefied by the addition of alginate-lyase (Fig. 1E). Despite clear differences in c-di-GMP levels and alginate production, we did not observe changes in biofilm structures in SCV24 when grown as biofilms in 96-well plates compared to its revertants (Fig. 1F). This suggests that the elevated c-di-GMP levels in SCV24 are produced by cyclases whose activity does not affect biofilm formation, as has been demonstrated for many cyclases (35).

### Complementation with functional *gacA* and *gacS* genes in the revertants restores the SCV phenotype but does not influence c-di-GMP levels.

**FIG 2:**
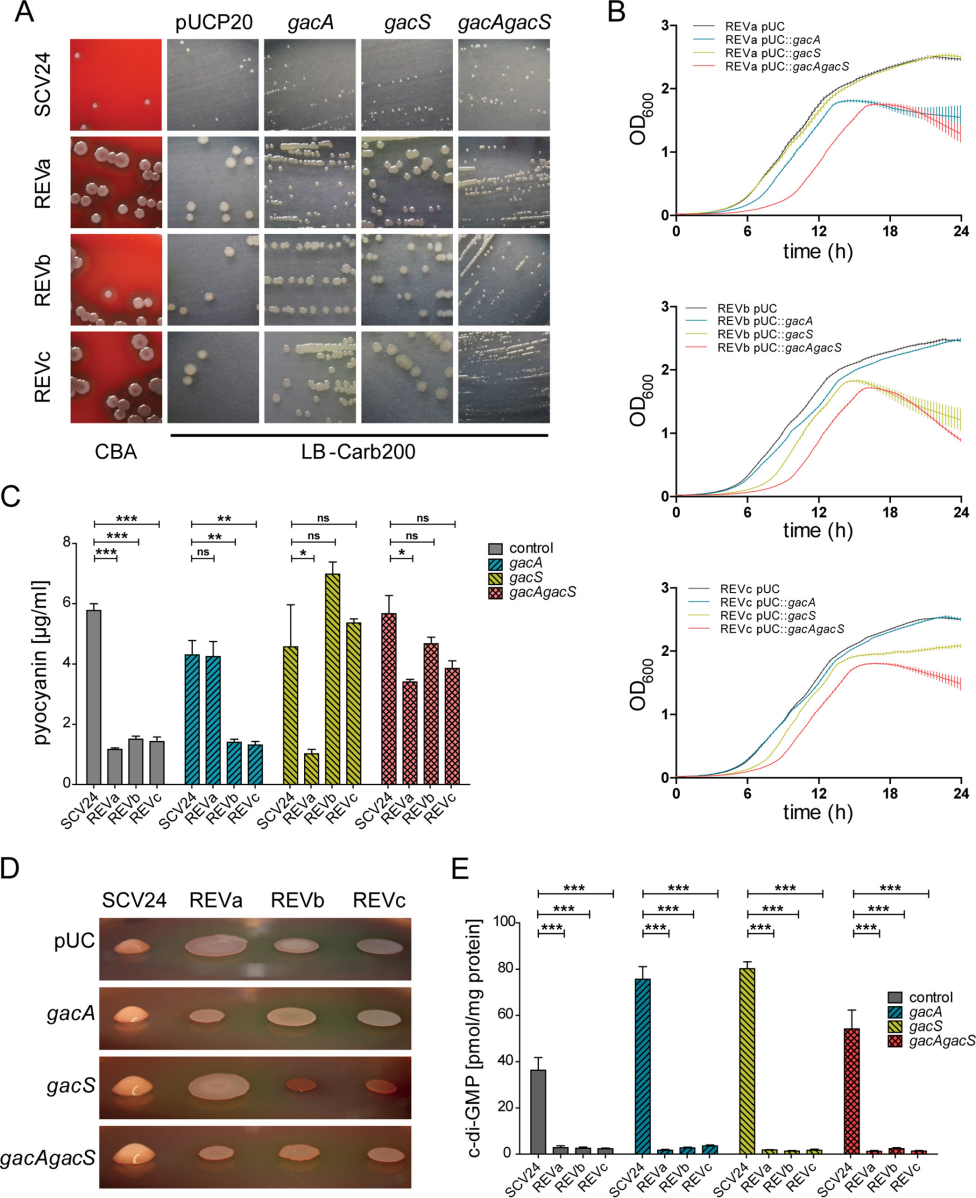
Complementation with functional *gacA* and *gacS* restores SCV24 phenotype only partially. (A) Overnight cultures were streaked on LB agar plates, and colony morphologies were recorded after 48 h of incubation (37°C). The leftmost panel depicts colony morphologies of the strains on Columbia blood agar (CBA) (SCV24, REVa exhibiting a stop mutation in *gacA*, REVb exhibiting a stop mutation in *gacS*, and REVc exhibiting a frameshift mutation within *gacS*). Functional *gacA*, *gacS*, or both *gacA* and *gacS* genes were introduced into the respective strains; the empty vector (pUCP20) served as a control. All pictures are shown at the same scale. (B) Wild-type *gacA* and *gacS* genes were introduced singly and in combination into the three SCV24 revertants on a pUCP20 vector, and growth curves were recorded in four biological replicates using a Bioscreen device. The average OD_600_ values are shown, and error bars represent the standard deviation. (C) Quantification of pyocyanin production in planktonic cultures in LB after 24 h. (D) Colony morphologies on Congo red agar after incubation for 14 days at room temperature. (E) c-di-GMP concentrations were quantified with liquid chromatography-mass spectrometry after 24 h of growth in LB at 37°C. For Figure 2C and E levels of statistical significance were calculated using a two-tailed unpaired *t* test (***, *P* < 0.001; **, *P* < 0.01; *, *P* < 0.1; n.s., not significant).

To provide experimental evidence that inactivation of *gacA* (in REVa) and/or *gacS* (in REVb and REVc) is linked to the SCV phenotype, we overexpressed *gacA*, *gacS*, and *gacA gacS* in the three revertant strain backgrounds. Indeed, overexpression of *gacA* in REVa (stop mutation in *gacA*) led to a switch to a small-colony phenotype comparable to that of SCV24 (Fig. 2A). While *gacS* expression alone was not sufficient to revert the colony morphology of REVb (stop mutation in *gacS*) and REVc (frameshift in *gacS*) to an SCV phenotype, this was the case if *gacA* and *gacS* were overexpressed simultaneously.

*gacA* overexpression in the REVa background and *gacS* and/or *gacA gacS* overexpression in the REVb and REVc background revealed an extended lag phase while growing in liquid LB cultures (Fig. 2B) and complemented pyocyanin production (Fig. 2C). However, alginate expression was not induced to the levels of SCV24 by overexpression of *gacA* and/or *gacS* in the revertants (Fig. 2D). In addition, although the c-di-GMP levels were clearly reduced in all three revertants, complementation of Gac/Rsm signaling in the revertants via overexpression of *gacA* and/or *gacS* did not restore the elevated c-di-GMP levels of SCV24 (Fig. 2E). Thus, although we were able to explain the faster growth and the revertant colony morphology, as well as the low pyocyanin production, in the revertants by an inactive Gac/Rsm signaling system, the differences in the activity of the system did not explain the high c-di-GMP and alginate production levels of SCV24.

### The transcriptional profile of the revertants reflects inactivation of the Gac/Rsm signaling pathway.

**FIG 3:**
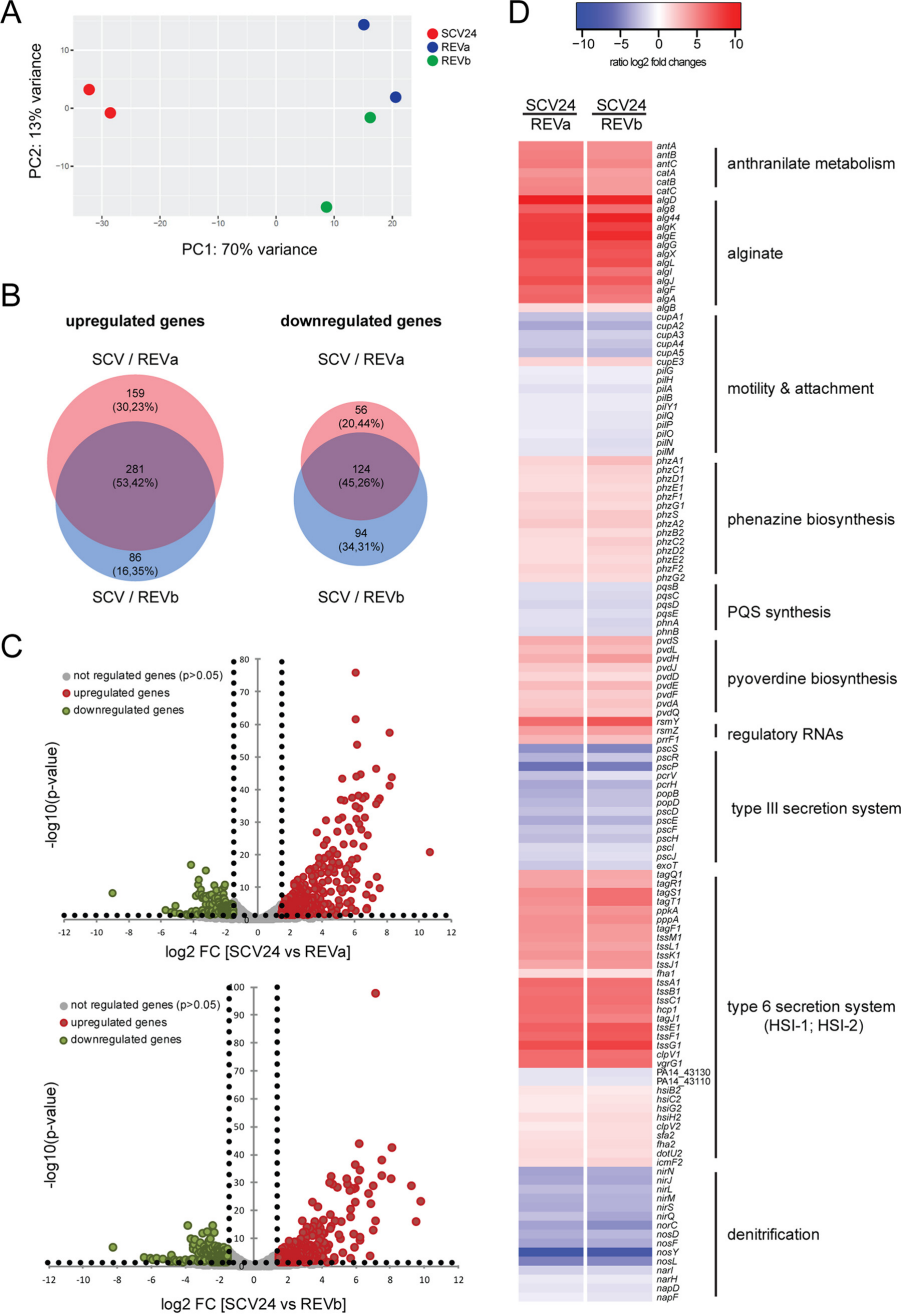
Transcriptome analysis uncovered differentially expressed genes in SCV24 in comparison to its respective revertants. (A) The clinical small-colony variant (SCV24) and its independently generated revertants (REVa and REVb) were subjected to RNA sequencing. A principal-component analysis shows a clear separation of the transcriptional profiles of SCV24 and its respective revertants. (B) Venn diagram depicting up- or downregulated genes (log_2_ fold change [FC] ≥ 1.5; log_2_ FC ≤ −1.5) of SCV24 compared to its REVa and REVb revertants (BioVenn). (C) Volcano plot of up- and downregulated genes in SCV24 compared to REVa (upper panel) or REVb (lower panel). The volcano plot shows the log_2_ fold difference (*x* axis) and the corresponding −log_10_(*P* values) (*y* axis) of regulated RNAs. Green circles represent mRNAs which were significantly less abundant, and red circles represent mRNAs which were significantly more abundant, in SCV24 (log_2_ FC ≥ 1.5; log_2_ FC ≤ −1.5) than in the respective revertants (*t* test *P* value ≤ 0.05). (D) Heatmap of selected differentially regulated genes and operons. PQS, Pseudomonas quinolone signal.

Next, we performed mRNA sequencing and analyzed the transcriptional profile of SCV24 compared to that of two independent revertants: REVa (stop codon in *gacA*) and REVb (stop codon in *gacS*). A principal-component analysis (PCA) demonstrates that the transcriptional profiles of SCV24 and the two revertants are clearly separate from each other (Fig. 3A). A total of 620 and 585 genes were differentially regulated (|log_2_ fold change| of >1.5; *P* < 0.05) in SCV24 compared to revertant REVa and REVb, respectively (Data Set S1). There was an overlap of 405 differentially regulated genes in the two comparisons (Fig. 3B; Table S4). The majority of genes were expressed at higher levels in SCV24 (Fig. 3B and C).

As expected, many genes of the *gacSA* regulon were found to be differentially regulated. Among these, the regulatory genes *rsmY* and *rsmZ* were most strongly upregulated in SCV24 compared to the revertants. Furthermore, genes encoding hydrogen cyanide (*hcn*), pyocyanin (*phz*), and the type VI secretion system (T6SS) HSI-I (e.g., *tss* and *tag*) were expressed at higher levels in SCV24. On the other hand, genes encoding components of the type III secretion system (T3SS), previously shown to be negatively affected by the Gac/Rsm regulon, were expressed at lower levels in SCV24 (Fig. 3D).

In accordance with the mucoid phenotype, we also found that the alginate genes were among the most strongly upregulated genes in SCV24 compared to its revertants. In P. aeruginosa alginate expression has not been described to be influenced by the *gacSA* regulon. However, a link between alginate expression and the Gac system has been demonstrated in Azotobacter vinelandii and Pseudomonas brassicacearum (36, 37). Furthermore, genes involved in pyoverdine biosynthesis (including the well-studied sigma factor *pvdS*) and in anthranilate metabolism, as well as the noncoding regulatory gene *prrf1*, were upregulated in SCV24 (Fig. 3D). Among the downregulated genes, we found *nir*, *nor*, *nos* (denitrification), *pqs* (quorum sensing), and *cup* (fimbrial biosynthesis) (Fig. 3D). The PseudoCAP categories “secreted factors” (upregulated in SCV24) and “energy metabolism” (downregulated in SCV24) were most strongly enriched within the differentially expressed genes (Fig. S1).

### Loss of high c-di-GMP levels of SCV24 upon passaging is independent of the reversion to a large-colony morphology.

**FIG 4:**
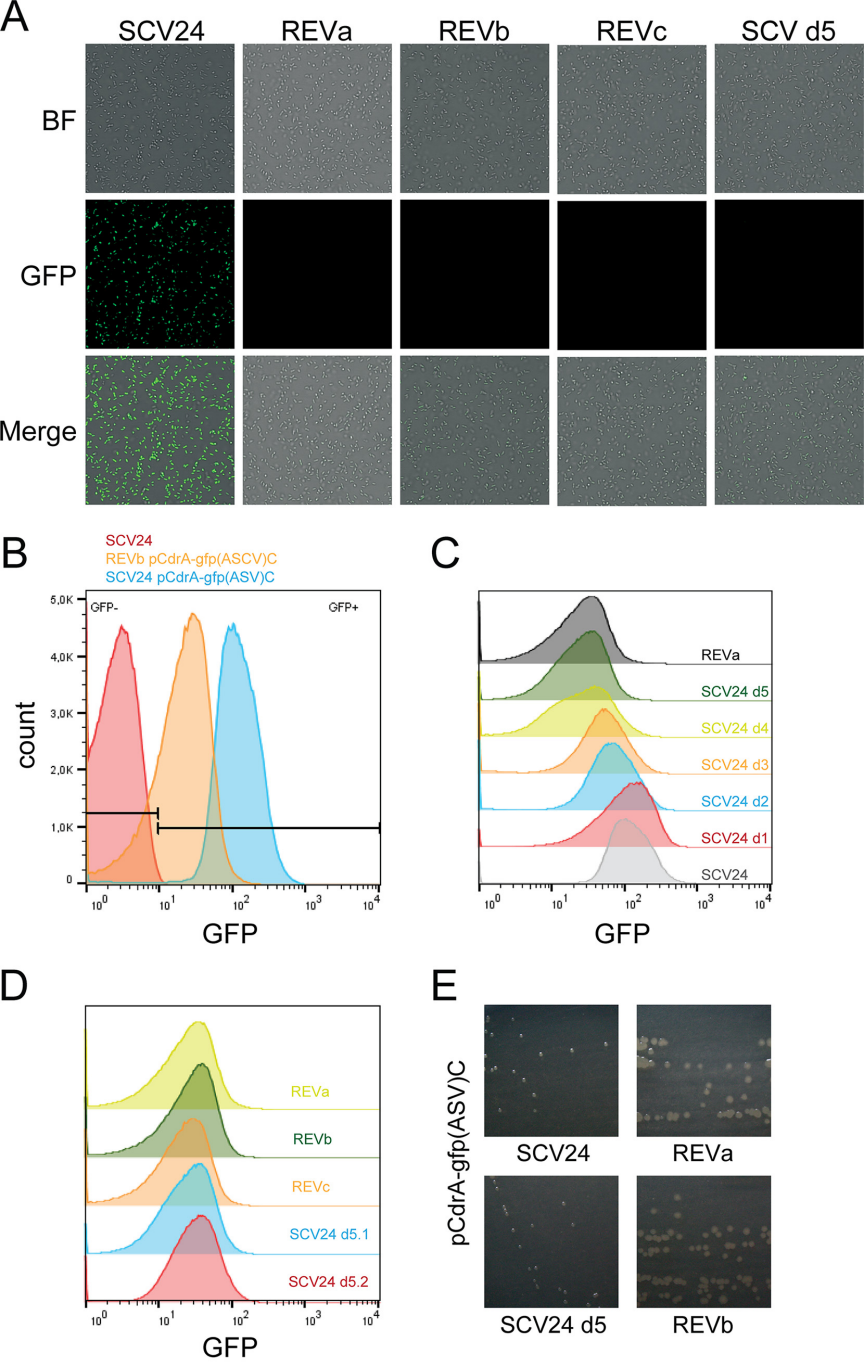
A fluorescence-based reporter reveals a stepwise decrease in the intracellular c-di-GMP levels. (A) The pCdrA-*gfp*(ASV)^C^ reporter was introduced into SCV24 and its three revertants as well as into SCV24 that had been passaged for 5 days. Upon a 5-day passage, SCV24 still retained the SCV phenotype. The density of bacteria from 2-day-old colonies suspended in PBS was monitored using bright-field (BF) microscopy (upper panel); fluorescence microscopy pictures of the same sample and merged images are shown in the middle and lower panels, respectively. (B) Differences in fluorescence intensities between SCV24 and its revertant REVb carrying the c-di-GMP reporter pCdrA-*gfp*(ASV)^C^ were monitored by flow cytometry. SCV24 without the vector served as a control. (C) Fluorescence of SCV24 pCdrA-*gfp*(ASV)^C^ was monitored once a day upon passaging under rich medium conditions over a period of 5 days (d1 to d5). Fluorescence intensities of the starting culture [SCV24 pCdrA-*gfp*(ASV)^C^] and the revertant strain [REVa pCdrA-*gfp*(ASV)^C^] were recorded as a control. (D) Fluorescence of the three revertants (REVa, REVb, and REVc) and two independently passaged SCV24 cultures (day 5) is depicted. (E) Representative photographs of the colony morphology of the small-colony variant (SCV24), its respective revertants (REVa and REVb), and the evolved SCV (SCV24 d5) grown for 48 h on LB plates at 37°C are shown.

We next aimed at investigating the loss of the high c-di-GMP levels in the three revertants. We used a transcriptional fusion of the c-di-GMP-responsive *cdrA* promoter to an unstable green fluorescent protein (GFP) reporter [*gfp*(ASV)] in order to monitor the intracellular c-di-GMP concentrations. First, we confirmed the high levels of c-di-GMP in SCV24 and demonstrated using fluorescence microscopy that the revertants exhibited lower c-di-GMP levels than SCV24 (Fig. 4A and B). Next, we used the reporter construct to monitor the c-di-GMP levels in SCV24 over time. We observed a stepwise decrease in fluorescence intensity during the course of passaging SCV24 under rich medium (LB) conditions (Fig. 4C). After 5 days of cultivation, SCV24 exhibited fluorescence intensities that were comparable to those found in the revertants (Fig. 4D). Strikingly, although the c-di-GMP concentration of the passaged SCV24 was already decreased to the revertant level after 5 days of passaging, the colony morphology was still small (Fig. 4E). The SCV24 colony morphology did not revert to large colonies before at least 7 days of passaging (overall 14 subcultures).

We next sequenced the *gacA* and *gacS* genes of SCV24, which had been passaged for 5 days (exhibiting an SCV phenotype but low levels of c-di-GMP), and could not detect any sequence alterations. These results clearly indicate that the loss of the high c-di-GMP levels of SCV24 is independent of the reversion to a large-colony morphology and is not associated with a loss in Gac/Rsm signaling.

### Daily transcriptome profiling of SCV24 passages exhibiting stepwise c-di-GMP level decreases.

Transcriptional profiling of two independent large-colony variant revertants (REVa and REVb) revealed an overlap of more than 400 genes that were differentially regulated compared to the parental SCV24 (Fig. 3D). In order to determine which of these genes were differentially regulated due to the inactivation of the Gac/Rsm system, and which due to the loss of c-di-GMP, we passaged SCV24 again for overall 7 days (seven subcultures) but now recorded the transcriptional profiles on a daily basis (Data Set S2). Of note, SCV24 retained the small-colony variant phenotype throughout all passages.

**FIG 5:**
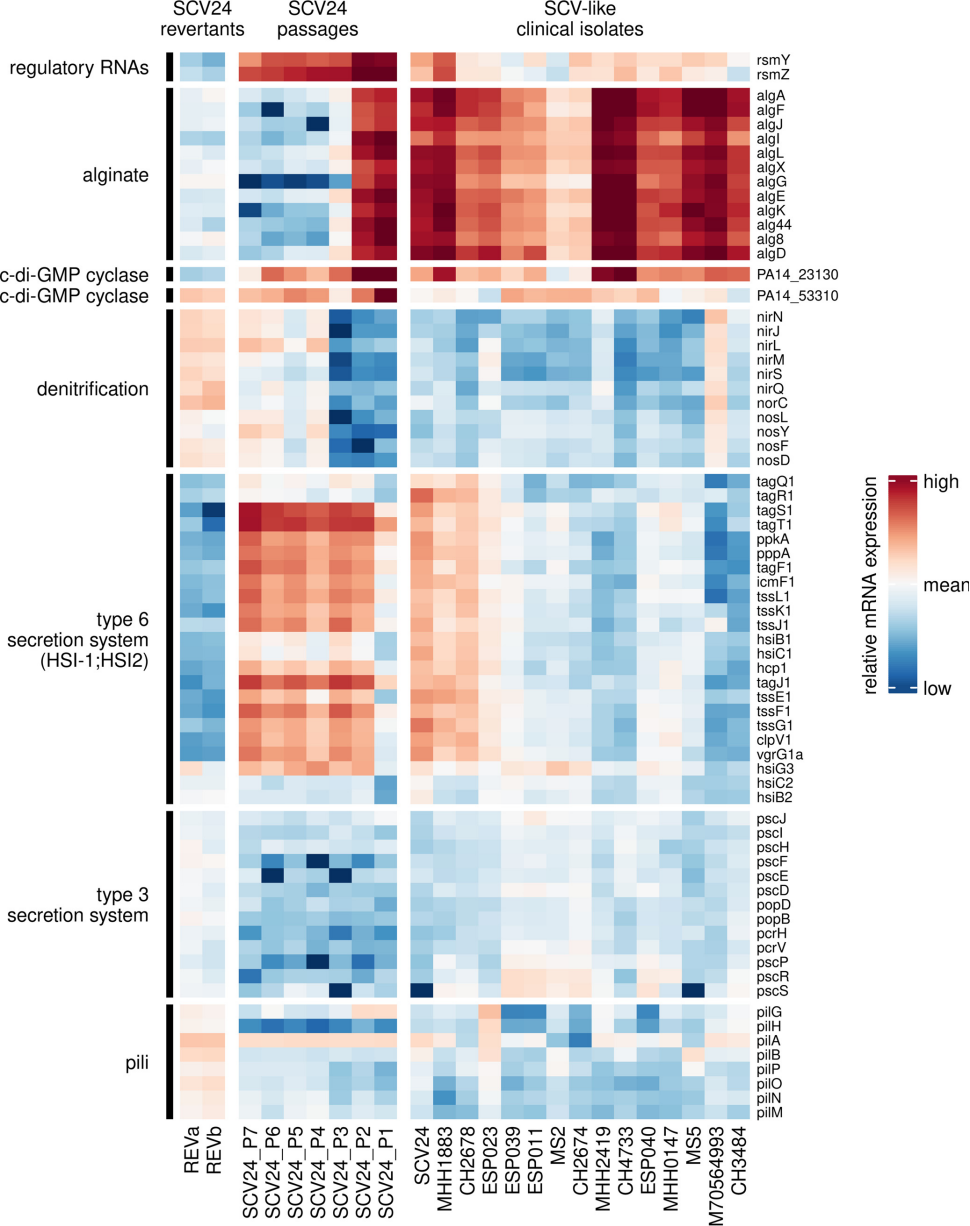
Heatmap of relative mRNA expression values of selected genes. The left panel depicts the mRNA levels of the SCV24 daily passages and the revertants compared to the levels of all 414 P. aeruginosa clinical isolates, whose transcriptional profiles have been previously recorded (39). The right panel shows the relative gene expression levels of the SCV-like clinical isolates including SCV24.

We performed a cluster analysis of the transcriptional profiles from the seven passages in order to identify patterns in the expression of individual genes over the course of passaging (Data Set S3). In total, we identified eight clusters (Fig. S2). In two clusters gene expression decreased over time (clusters 1 and 2), while in three other clusters gene expression increased (clusters 3 to 5). Clusters 1 and 2 included the regulatory RNAs *rsmY* and *rsmZ* and the majority of the alginate genes, which exhibited a stepwise decrease in their expression level. While the alginate genes reached the expression level of the revertants (REVa and REVb), the expression levels of *rsmY* and *rsmZ* did not quite reach the low levels of the revertants (Fig. 5, left panel). Interestingly, we found two genes harboring a GGDEF domain necessary for c-di-GMP production: PA14_23130 in cluster 1 and PA14_53310 in cluster 2. Both genes showed a gradual decrease in their expression level over the passages until they reached the expression level of the revertants. Three other clusters contained genes whose expression gradually increased from passage 1 to passage 7. In one of them (cluster 3), *nir*, *nor*, and *nos* were present. The expression levels at the end of the 7-day passaging were comparable to those of the revertants (REVa and REVb).

As expected, we identified genes that did not show a gradual change in their expression levels during the 7-day passaging but were affected by inactivation of the Gac/Rsm system (Fig. 5, left panel). These included genes belonging to the T6SS and T3SS and genes important for pilus-related motility.

### Correlation of the small-colony variant phenotype and gene expression data of clinical isolates.

**FIG 6:**
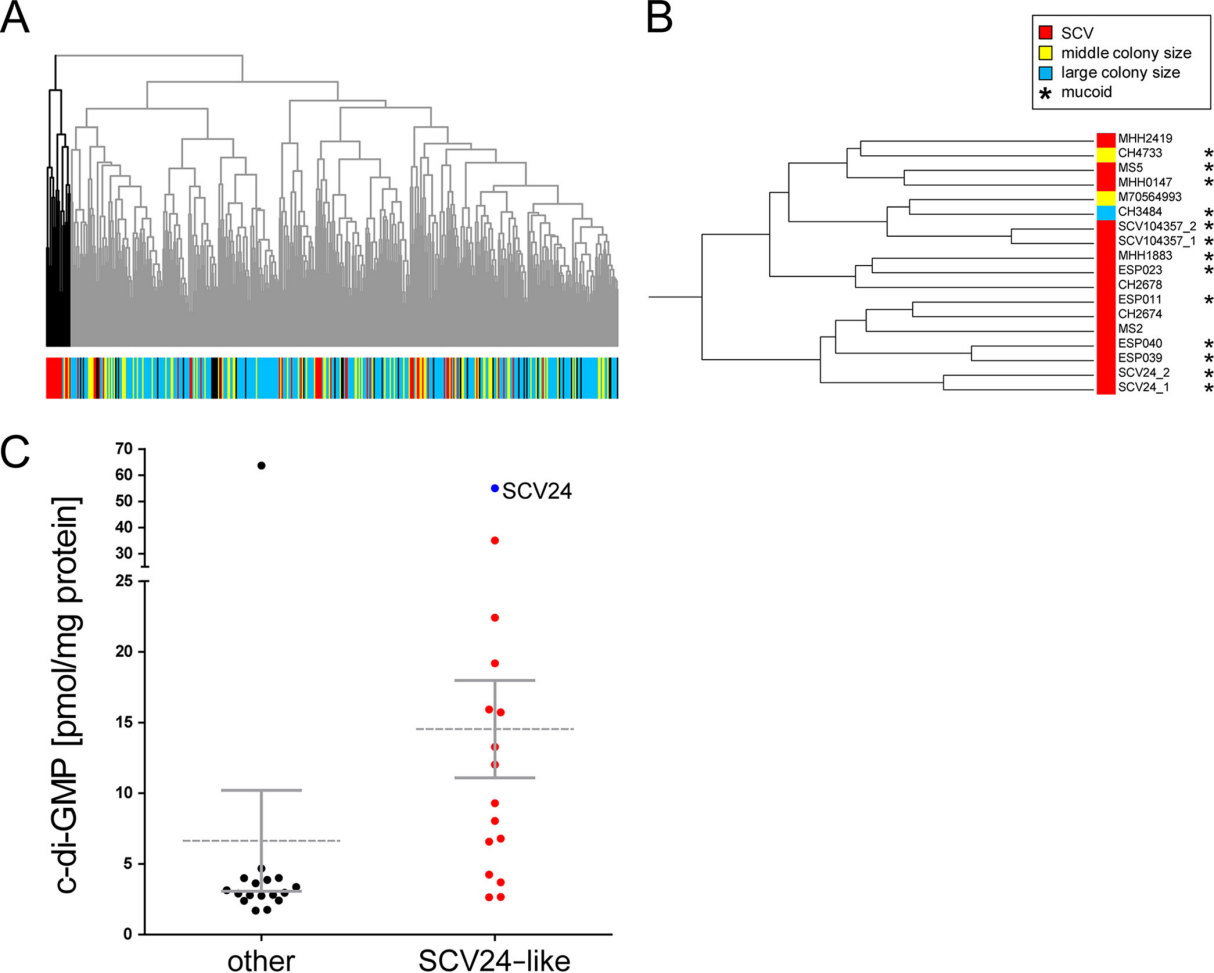
Gene expression clustering of clinical isolates. (A) The dendrogram is the result of a hierarchical clustering analysis derived from a Euclidean distance matrix that is based on a nonparametric rank-based ordering of expression profiles of those 405 genes that were found to be commonly differentially regulated (|log_2_ FC| ≥ 1.5) in SCV24 in comparison to its two revertants, REVa and REVb, across all analyzed clinical isolates, including SCV24. The color code reflects the colony size of the clinical isolates from red, very small (SCV), to yellow, blue, and black with increasing colony size (for further information, see Materials and Methods). (B) Close-up of the smaller cluster (bold section on the left in panel A) that contained SCV24, which grouped with 15 additional clinical isolates. Mucoidy is indicated by asterisks. (C) c-di-GMP levels as determined by liquid chromatography-mass spectrometry of the 15 SCV24-like isolates (red) and SCV24 (blue) compared to 16 randomly selected clinical isolates.

Next, we aimed at exploring whether the phenotype of SCV24 is unique or whether the phenotype is shared by other clinical P. aeruginosa isolates. We made use of our collection of clinical isolates, for which transcriptional profiles (38) and colony morphologies on agar plates (39) have been recorded. As can be seen in Fig. 5 (right panel) and Fig. 6A, the gene expression status of SCV24 clustered together with 15 additional clinical P. aeruginosa isolates. Fourteen of those 16 strains had an SCV phenotype, 10 of which were also mucoid (Fig. 6B; Table S5). We also determined c-di-GMP levels of SCV24-like isolates. In general, they produced higher levels of the intracellular second messenger c-di-GMP than did 16 randomly selected isolates of the larger cluster (Fig. 6C).

Of note, in most of the 16 clinical isolates many cluster 1 to 5 genes were expressed at a similar level as in SCV24. These included *rsmY* and *rsmZ*, which exhibited at least a 15-fold-higher expression in the SCV24-like clinical isolates than the average expression of our collection of 414 clinical isolates (Fig. S3). Furthermore, the PA14_23130 gene encoding a GGDEF-containing protein and the coregulated alginate genes were expressed at a higher level in the clinical SCV-like isolates (Fig. 5). This indicates that in SCV24-like phenotypes, which seem to have repeatedly evolved in clinical isolates, this cyclase might be responsible for the elevated levels of c-di-GMP to drive alginate production.

## DISCUSSION

If not eradicated during acute infections, P. aeruginosa populations can cause chronic infections, where the bacteria persist and evolve (40). With progression and chronicity of the disease, infected host sites become more heterogeneous and trigger the recovery of morphologically diverse bacterial phenotypes as a typical hallmark (41). Those clinical isolates have gone through adaptive evolution and have acquired mutations that reflect bacterial adaptation to the habitat of the human host (7, 42). The study of adapted strains thus provides a unique opportunity to associate the presence of adaptive mutations with the activation of specific regulatory processes and thus to gain information on the molecular mechanisms underlying phenotypes that facilitate survival during infections.

It has been demonstrated previously that mutations affecting the activity of key regulators of c-di-GMP production drive the formation of the SCV phenotype (25, 26, 43). As a result, many clinical SCVs exhibit biofilm-associated phenotypes, even under non-biofilm-inducing environmental conditions. In this study, we isolated fast-growing revertants of a clinical SCV, which exhibited high c-di-GMP levels and increased alginate production, and by applying a whole-genome sequencing approach, we identified exclusively inactivating mutations in either the sensor (GacS) or the response regulator (GacA) as being responsible for the phenotypic switch to a large revertant colony morphology. The activated GacS/GacA two-component system in SCV24 resulted in increased levels of the downstream small regulatory RNA targets *rsmY* and *rsmZ.* Those two small RNAs sequester the RNA-binding protein RsmA to relieve repression of downstream-regulated genes and thus enhance secondary metabolism, such as the production of pyocyanin (44). Of note, the GacS/GacA two-component system and its downstream small regulatory RNA targets have been shown before to be important for the global control of genes associated with acute and chronic infections (45). RsmA represses synthesis of the exopolysaccharides Pel and Psl, quorum sensing, and genes encoding the type VI secretion system (T6SS), whereas RsmA promotes T2SS, T3SS, swarming, and the production of type IV pili (32, 46). In general, it seems that phenotypes that have been associated with the SCV morphology (e.g., hyperpiliation, increased antibiotic resistance, and biofilm formation [14]) require an intact Gac/Rsm system, while pathways activated by high RsmA expression are frequently downregulated in SCVs. Of note, SCV24 as well as 15 additional clinical isolates exhibited an at least 15-fold-higher expression of the *rsmY* and *rsmZ* genes than the average expression of our collection of 414 clinical isolates, and 14 of them exhibited an SCV phenotype (see Fig. S3 in the supplemental material).

In accordance with our results, Irie et al. showed that a dysfunction of the Gac/Rsm signaling cascade due to a deletion of *rsmA* inhibits phenotypes associated with acute infections and results in the induction of an SCV phenotype in P. aeruginosa (46). Furthermore, spontaneous mutations in *gacS* and/or *gacA* are known to occur frequently also in other Pseudomonas species. Mutations in the GacSA system have been observed under laboratory conditions as well as in the plant rhizosphere and affect the virulence and colony morphology phenotype of phytopathogens, such as Pseudomonas fluorescens and P. brassicacearum (37, 47, 48).

It was tempting to assume that the high c-di-GMP levels in SCV24 were due to an active Gac/Rsm signaling pathway. A direct link between the Gac/Rsm pathway and c-di-GMP signaling has been reported in several bacteria. The homologous GacS/GacA two-component systems in E. coli (BarA/UvrY) and Salmonella enterica (BarA/SirA) modulate the activity of the translational repressor CsrA, via the activation of two small regulatory RNAs, *csrB* and *csrC*, to regulate genes encoding c-di-GMP-modulating proteins (harboring GGDEF, GGDEF/EAL, or EAL domains) (49, 50). In Xanthomonas campestris, RsmA has been shown to control three GGDEF domain proteins posttranscriptionally (51). Furthermore, the Filloux laboratory reported on the link between the GacSA system and c-di-GMP signaling in P. aeruginosa. They observed that the diguanylate cyclase SadC is active in a *retS* mutant and responsible for induced biofilm formation and increased c-di-GMP levels (52, 53). In general, RetS acts as an inhibitor and forms heterodimers with GacS, thus preventing autophosphorylation of GacS and activation of the cognate response regulator GacA, which triggers the Gac/Rsm cascade (54). This in turn leads to a repression of SadC, thereby inhibiting biofilm production and c-di-GMP levels. Nevertheless, it is still an open question whether there is a direct link of the regulation of SadC by RsmA or whether the Gac/Rsm system and c-di-GMP signaling are connected indirectly.

The results of our study confirm the close connection of the Gac/Rsm signaling and c-di-GMP levels. Not only in SCV24 but also in 15 additional clinical SCV24-like isolates, we found a highly active Gac/Rsm signaling system that was positively correlated with high c-di-GMP levels and a mucoid phenotype. However, we also found that the loss of high c-di-GMP levels and mucoidy was independent of the Gac/Rsm-mediated SCV24 colony morphology switch. While the switching of colony morphology occurred later during reversion and directly relied on the activity of the GacSA system, loss of the high c-di-GMP levels and alginate overproduction occurred earlier and before the acquisition of the GacSA inactivating mutations in the revertants.

In addition to production of SCV phenotypes, the conversion to a mucoid phenotype is a hallmark of P. aeruginosa isolates recovered from the lungs of chronically infected CF patients (6). Alginate-overproducing variants arise frequently due to mutations in the anti-sigma factor *mucA*. These mutations impair the binding and inhibition of the alternative sigma factor AlgU, which induces transcription of the *alg* operon (6, 13). In addition, alginate biosynthesis was shown to be posttranslationally regulated by c-di-GMP (31). Alg44, an inner membrane protein encoded within the *alg* operon, contains a PilZ domain that binds c-di-GMP to induce alginate production (55). Interestingly, in SCV24 we found a high level of c-di-GMP and a nonsense mutation in *mucA* (Gln117*), both of which could explain the mucoid phenotype of SCV24 (Table S5). However, while the revertants lost their mucoid phenotypes, albeit they still harbored the *mucA* mutation, we observed a correlation between decreasing c-di-GMP levels and gradual loss of alginate biosynthetic gene expression during the early passages of SCV24. This suggest that there is a causal relationship between increased c-di-GMP levels and alginate production. It is tempting to speculate that the PA14_23130 gene, encoding a GGDEF domain protein, is involved in the provision of respective c-di-GMP levels that drive the expression of alginate biosynthesis genes not only in SCV24 but in other SCV24-like clinical isolates that express high levels of alginate biosynthesis genes. A transcriptional regulation of alginate biosynthesis by c-di-GMP has been demonstrated before (56).

In conclusion, it seems that high c-di-GMP levels of SCV24 have been induced in the unique environment of the infected human host and those levels remain elevated for prolonged periods, even if the bacteria were cultivated under noninducing conditions (57). This lagging of the reduction of c-di-GMP levels upon a switch to noninducing conditions is reminiscent of a hysteretic response. Hysteresis is the dependence of the state of a system on its history and allows for a transition of states that is less susceptible to noise. Although planktonic growth in a rich medium as an input should result in low c-di-GMP levels as an output, SCV24 keeps producing higher levels of c-di-GMP for prolonged periods. Interestingly, both high levels of c-di-GMP and active Gac/Rsm signaling induce similar and in large parts overlapping phenotypes, so that the hysteretic c-di-GMP-mediated response could provide the unique opportunity to stabilize a short-term individual transcriptional regulation of biofilm-associated genes, before a genetic modulation, e.g., of the activity of the Gac/Rsm system, guarantees long-term evolutionary adaptation (58).

## MATERIALS AND METHODS

### Strains and growth conditions.

Bacterial strains and plasmids used in this study are listed in Table S2 in the supplemental material. Unless otherwise indicated, all P. aeruginosa and E. coli strains were grown in lysogeny broth (LB; 5 g/L yeast extract, 7.5 g/L NaCl, 10 g/L tryptone) at 37°C with shaking (180 rpm) or on agar plates (LB solidified with 1.6% [wt/vol] agar).

To generate revertants *in vitro*, independent precultures from SCV24 were inoculated in 3 mL of fresh LB with a starting OD_600_ of 0.01. The bacteria were transferred twice daily when turbidity was visible to a new culture with a starting OD_600_ of 0.01 (evening) or 0.05 (morning). In parallel, an aliquot of each culture was streaked on Columbia blood agar plates (Becton, Dickinson) to monitor for reversion to large surface colonies. This process was repeated until a stable revertant was generated (1 to 2 weeks).

Images of the colony morphology and transcriptional profiles under planktonic conditions (LB medium OD_600_ of 2) of the 414 clinical isolates are publicly available in the interactive database BACTOME (38, 39, 59).

### Plasmid and strain construction.

The *gacA*, *gacS*, and *gacA gacS* genes were amplified by PCR using primers listed in Table S3. PCR products were inserted into the shuttle vector pUCP20 linearized with EcoRI and XbaI (*gacA*) or XbaI and HindIII (*gacS*). The plasmid pCdrA-*gfp*(ASV)^C^ was purchased from Addgene (plasmid no. 111615) (60).

### DNA extraction and whole-genome sequencing.

Bacterial DNA was extracted using the DNeasy Blood and Tissue kit (Qiagen) and fragmented using the S2/E210 focused ultrasonicator (Covaris). Using the NEBNext Ultra DNA library prep kit for Illumina (New England Biolabs [NEB]), a library was prepared and sequenced in paired-end mode (2 × 150-bp reads) on an Illumina HiSeq 2500 device. Clipped reads were mapped to the UCBPP-PA14 reference genome. For SNP detection, SAMtools (v.0.1.19) and Parsnp (v.1.2) were used with the PA14 genome as a reference. Every identified SNP position was rechecked manually in the Integrative Genome Viewer (IGV) (v.2.3.98).

### RNA sequencing and data analysis.

Bacteria were grown in 10 mL LB to early stationary phase (OD_600_ of 2, 37°C, 180 rpm). RNA was extracted using the RNeasy minikit (Qiagen) and QIAshredder columns (Qiagen). rRNA was removed by the Ribo-Zero bacterial kit (Illumina), and cDNA libraries were generated with the ScriptSeq v2 kit (Illumina). The samples were sequenced in single-end mode on an Illumina HiSeq 2500 device (1 × 50-bp reads). Mapping was performed using a Stampy pipeline (61) with the UCBPP-PA14 genome as a reference. Differential gene expression analysis and PCA were performed with the R package DESeq2 (v.1.18.1) (62).

For differential gene expression analyses, a log_2_ fold change of 1.5 was used as the cutoff value (with *P* of <0.05). To allow for a nonparametric analysis, the reads for each gene were transformed into ranks. The rank abundance values were used to create a dendrogram based on an automated hierarchical clustering analysis derived from a Euclidean distance matrix using default settings in the R functions dist and hclust (v.3.6.3). Three-dimensional (3D) scatterplots were designed with the R package plot3D (v.1.3). The depicted alginate expression level is an arbitrary unit and is based on the ratio of the mean normalized expression of the 12 structural genes in the *alg* operon (*algA*, -*D*, -*E*, -*F*, -*G*, -*I*, -*J*, -*K*, -*L*, -*X*, -*8*, and -*44*) for each strain against the mean normalized expression of the *alg* operon genes of the reference strain PA14. Similarly, the *rsmY/Z* gene expression is based on the ratio of the mean normalized expression of *rsmY* and *rsmZ* to the mean normalized expression of the *rsmY/Z* genes of the reference strain PA14. The correlation matrix was calculated using the corrplot package (v.0.84) with default settings. Area proportional Venn diagrams were generated using the online tool BioVenn (63).

### Pyocyanin production.

Pyocyanin production was analyzed as previously described (64). Briefly, pyocyanin was extracted by the addition of an equal volume of chloroform to a bacterial culture. Three milliliters of the organic phase was mixed with 1 mL 0.2 M hydrochloric acid (HCl), and the absorbance was determined at 520 nm for the aqueous phase. Pyocyanin concentrations (micrograms per milliliter of supernatant) were calculated by multiplication with the correction factor 17.072.

### Extraction and quantification of cyclic di-GMP levels.

The extraction of c-di-GMP and its quantification by high-performance liquid chromatography-coupled tandem mass spectrometry were performed as described previously with some modifications (65, 66). Briefly, c-di-GMP was extracted with methanol-acetonitrile-water (2:2:1) from 5 mL of a bacterial culture grown for 24 h in LB at 37°C. Isotope-labeled [^13^C, ^15^N]c-di-GMP was used as internal standard.

### Biofilm formation and confocal microscopy.

Altered biofilm characteristics between SCVs and their corresponding revertants were monitored by confocal microscopy as previously described (67), and fluorescence of the c-di-GMP reporter pCdrA-*gfp*(ASV)^C^ was monitored following the resuspension of agar-grown single colonies grown in 0.9% saline.

### Flow cytometry-based c-di-GMP measurements.

To monitor c-di-GMP levels in P. aeruginosa cells during the *in vitro* generation of revertants, strains containing the plasmid pCdrA::*gfp*(ASV)^C^ were cultured overnight in LB medium supplemented with 200 μg/mL carbenicillin. Bacteria were harvested by centrifugation at 8,000 × *g* for 3 min, washed, and resuspended in 1 mL 1× phosphate-buffered saline (PBS) twice. This cell suspension was diluted 1:5 in PBS and fixed with 2% paraformaldehyde at room temperature (RT) for 1.5 h. c-di-GMP levels were determined by measuring GFP intensity on a FACSCalibur (BD Pharmingen; BD CellQuest Pro 4.0.2 software) and analyzed by FlowJo V10 software (FlowJo, LLC).

### Daily transcriptome profiling of SCV24 passages.

SCV24 was passaged in rich LB medium for seven times overall, and bacteria from each passage were harvested for RNA sequencing at an OD_600_ of 2 (in duplicates). cDNA libraries were prepared as previously described (68). Libraries were sequenced on an Illumina NovaSeq 6000 (paired-end mode; 2 × 50 bp). Raw reads were trimmed using the tool Cutadapt (version 3.5) (69) with customized settings (--nextseq-trim = 20; -g ‘TTTTT;min_overlap = 1’; -G ‘GGGGGGG;min_overlap = 3’; -m 36). Mapping was performed with Bowtie2 (version 2.3.5.1) (70) with the settings “--very-sensitive-local; --no-mixed; --fr; --no-unal” and with the UCBPP-PA14 genome as a reference. Reads per gene were extracted with the tool featureCounts (version 2.0.1) (71). Normalized reads per gene were calculated with the R package edgeR (v.3.32.1) (72). Differential gene expression was performed with the R package DESeq2 (v.1.18.1). Cluster analysis was performed using R package Mfuzz (v2.52.0) with log_2_ normalized reads per gene as input. All differentially expressed genes throughout the passages were clustered into 8 clusters [estimated by the Mfuzz:Dmin() function] (73).

### Data availability.

The transcriptional data were previously uploaded to the Gene Expression Omnibus (GEO) database with the accession number GSE123544, and for this study the transcriptional data of the SCVs and their revertants were uploaded with the accession number GSE147921. The genomic data of the 414 clinical isolates were previously uploaded to the Sequence Read Archive (SRA) with the accession number PRJNA526797, and the genomic data for the SCVs and their revertants were uploaded with the accession number PRJNA622425.

## References

[1] Vasil ML. 1986. *Pseudomonas aeruginosa*: biology, mechanisms of virulence, epidemiology. J Pediatr 108:800–805. doi:10.1016/s0022-3476(86)80748-x. 3009772

[2] Klockgether J, Tümmler B. 2017. Recent advances in understanding *Pseudomonas aeruginosa* as a pathogen. F1000Res 6:1261. doi:10.12688/f1000research.10506.1. 28794863 PMC5538032

[3] Gellatly SL, Hancock RE. 2013. *Pseudomonas aeruginosa*: new insights into pathogenesis and host defenses. Pathog Dis 67:159–173. doi:10.1111/2049-632X.12033. 23620179

[4] Römling U, Fiedler B, Bosshammer J, Grothues D, Greipel J, von der Hardt H, Tümmler B. 1994. Epidemiology of chronic *Pseudomonas aeruginosa* infections in cystic fibrosis. J Infect Dis 170:1616–1621. doi:10.1093/infdis/170.6.1616. 7996008

[5] Boles BR, Thoendel M, Singh PK. 2004. Self-generated diversity produces “insurance effects” in biofilm communities. Proc Natl Acad Sci USA 101:16630–16635. doi:10.1073/pnas.0407460101. 15546998 PMC528905

[6] Boucher JC, Yu H, Mudd MH, Deretic V. 1997. Mucoid *Pseudomonas aeruginosa* in cystic fibrosis: characterization of muc mutations in clinical isolates and analysis of clearance in a mouse model of respiratory infection. Infect Immun 65:3838–3846. doi:10.1128/iai.65.9.3838-3846.1997. 9284161 PMC175548

[7] Hogardt M, Heesemann J. 2010. Adaptation of *Pseudomonas aeruginosa* during persistence in the cystic fibrosis lung. Int J Med Microbiol 300:557–562. doi:10.1016/j.ijmm.2010.08.008. 20943439

[8] Price TD, Qvarnstrom A, Irwin DE. 2003. The role of phenotypic plasticity in driving genetic evolution. Proc Biol Sci 270:1433–1440. doi:10.1098/rspb.2003.2372. 12965006 PMC1691402

[9] Lyczak JB, Cannon CL, Pier GB. 2000. Establishment of *Pseudomonas aeruginosa* infection: lessons from a versatile opportunist. Microbes Infect 2:1051–1060. doi:10.1016/s1286-4579(00)01259-4. 10967285

[10] McCann KS. 2000. The diversity-stability debate. Nature 405:228–233. doi:10.1038/35012234. 10821283

[11] Yachi S, Loreau M. 1999. Biodiversity and ecosystem productivity in a fluctuating environment: the insurance hypothesis. Proc Natl Acad Sci USA 96:1463–1468. doi:10.1073/pnas.96.4.1463. 9990046 PMC15485

[12] Leid JG, Willson CJ, Shirtliff ME, Hassett DJ, Parsek MR, Jeffers AK. 2005. The exopolysaccharide alginate protects *Pseudomonas aeruginosa* biofilm bacteria from IFN-gamma-mediated macrophage killing. J Immunol 175:7512–7518. doi:10.4049/jimmunol.175.11.7512. 16301659

[13] Govan JR, Martin DW, Deretic VP. 1992. Mucoid *Pseudomonas aeruginosa* and cystic fibrosis: the role of mutations in muc loci. FEMS Microbiol Lett 100:323–329. doi:10.1111/j.1574-6968.1992.tb14059.x. 1478467

[14] Häussler S, Tümmler B, Weissbrodt H, Rohde M, Steinmetz I. 1999. Small-colony variants of *Pseudomonas aeruginosa* in cystic fibrosis. Clin Infect Dis 29:621–625. doi:10.1086/598644. 10530458

[15] Starkey M, Hickman JH, Ma L, Zhang N, De Long S, Hinz A, Palacios S, Manoil C, Kirisits MJ, Starner TD, Wozniak DJ, Harwood CS, Parsek MR. 2009. *Pseudomonas aeruginosa* rugose small-colony variants have adaptations that likely promote persistence in the cystic fibrosis lung. J Bacteriol 191:3492–3503. doi:10.1128/JB.00119-09. 19329647 PMC2681918

[16] Häussler S. 2004. Biofilm formation by the small colony variant phenotype of *Pseudomonas aeruginosa*. Environ Microbiol 6:546–551. doi:10.1111/j.1462-2920.2004.00618.x. 15142242

[17] Häußler S, Ziegler I, Löttel A, Götz FV, Rohde M, Wehmhöhner D, Saravanamuthu S, Tümmler B, Steinmetz I. 2003. Highly adherent small-colony variants of *Pseudomonas aeruginosa* in cystic fibrosis lung infection. J Med Microbiol 52:295–301. doi:10.1099/jmm.0.05069-0. 12676867

[18] Wei Q, Tarighi S, Dötsch A, Häussler S, Müsken M, Wright VJ, Camara M, Williams P, Haenen S, Boerjan B, Bogaerts A, Vierstraete E, Verleyen P, Schoofs L, Willaert R, De Groote VN, Michiels J, Vercammen K, Crabbe A, Cornelis P. 2011. Phenotypic and genome-wide analysis of an antibiotic-resistant small colony variant (SCV) of Pseudomonas aeruginosa. PLoS One 6:e29276. doi:10.1371/journal.pone.0029276. 22195037 PMC3240657

[19] Proctor RA, von Eiff C, Kahl BC, Becker K, McNamara P, Herrmann M, Peters G. 2006. Small colony variants: a pathogenic form of bacteria that facilitates persistent and recurrent infections. Nat Rev Microbiol 4:295–305. doi:10.1038/nrmicro1384. 16541137

[20] Malone JG, Jaeger T, Manfredi P, Dötsch A, Blanka A, Bos R, Cornelis GR, Häussler S, Jenal U. 2012. The YfiBNR signal transduction mechanism reveals novel targets for the evolution of persistent Pseudomonas aeruginosa in cystic fibrosis airways. PLoS Pathog 8:e1002760. doi:10.1371/journal.ppat.1002760. 22719254 PMC3375315

[21] Lim WS, Phang KK, Tan AH, Li SF, Ow DS. 2016. Small colony variants and single nucleotide variations in Pf1 region of PB1 phage-resistant *Pseudomonas aeruginosa*. Front Microbiol 7:282. doi:10.3389/fmicb.2016.00282. 27014207 PMC4783410

[22] Hickman JW, Tifrea DF, Harwood CS. 2005. A chemosensory system that regulates biofilm formation through modulation of cyclic diguanylate levels. Proc Natl Acad Sci USA 102:14422–14427. doi:10.1073/pnas.0507170102. 16186483 PMC1234902

[23] Malone JG, Jaeger T, Spangler C, Ritz D, Spang A, Arrieumerlou C, Kaever V, Landmann R, Jenal U. 2010. YfiBNR mediates cyclic di-GMP dependent small colony variant formation and persistence in *Pseudomonas aeruginosa*. PLoS Pathog 6:e1000804. doi:10.1371/journal.ppat.1000804. 20300602 PMC2837407

[24] Pitton M, Oberhaensli S, Appiah F, Pagani JL, Fournier A, Jakob SM, Que YA, Cameron DR. 2022. Mutation to *ispA* produces stable small-colony variants of *Pseudomonas aeruginosa* that have enhanced aminoglycoside resistance. Antimicrob Agents Chemother 66:e00621-22. doi:10.1128/aac.00621-22. 35852364 PMC9295567

[25] Drenkard E, Ausubel FM. 2002. Pseudomonas biofilm formation and antibiotic resistance are linked to phenotypic variation. Nature 416:740–743. doi:10.1038/416740a. 11961556

[26] Meissner A, Wild V, Simm R, Rohde M, Erck C, Bredenbruch F, Morr M, Römling U, Häussler S. 2007. *Pseudomonas aeruginosa cupA*-encoded fimbriae expression is regulated by a GGDEF and EAL domain-dependent modulation of the intracellular level of cyclic diguanylate. Environ Microbiol 9:2475–2485. doi:10.1111/j.1462-2920.2007.01366.x. 17803773

[27] Kirisits MJ, Prost L, Starkey M, Parsek MR. 2005. Characterization of colony morphology variants isolated from *Pseudomonas aeruginosa* biofilms. Appl Environ Microbiol 71:4809–4821. doi:10.1128/AEM.71.8.4809-4821.2005. 16085879 PMC1183349

[28] Christen B, Christen M, Paul R, Schmid F, Folcher M, Jenoe P, Meuwly M, Jenal U. 2006. Allosteric control of cyclic di-GMP signaling. J Biol Chem 281:32015–32024. doi:10.1074/jbc.M603589200. 16923812

[29] Hengge R. 2009. Principles of c-di-GMP signalling in bacteria. Nat Rev Microbiol 7:263–273. doi:10.1038/nrmicro2109. 19287449

[30] McDougald D, Rice SA, Barraud N, Steinberg PD, Kjelleberg S. 2011. Should we stay or should we go: mechanisms and ecological consequences for biofilm dispersal. Nat Rev Microbiol 10:39–50. doi:10.1038/nrmicro2695. 22120588

[31] Römling U, Galperin MY, Gomelsky M. 2013. Cyclic di-GMP: the first 25 years of a universal bacterial second messenger. Microbiol Mol Biol Rev 77:1–52. doi:10.1128/MMBR.00043-12. 23471616 PMC3591986

[32] Kay E, Humair B, Denervaud V, Riedel K, Spahr S, Eberl L, Valverde C, Haas D. 2006. Two GacA-dependent small RNAs modulate the quorum-sensing response in *Pseudomonas aeruginosa*. J Bacteriol 188:6026–6033. doi:10.1128/JB.00409-06. 16885472 PMC1540078

[33] Lo YL, Shen L, Chang CH, Bhuwan M, Chiu CH, Chang HY. 2016. Regulation of motility and phenazine pigment production by FliA is cyclic-di-GMP dependent in *Pseudomonas aeruginosa* PAO1. PLoS One 11:e0155397. doi:10.1371/journal.pone.0155397. 27175902 PMC4866697

[34] Okegbe C, Fields BL, Cole SJ, Beierschmitt C, Morgan CJ, Price-Whelan A, Stewart RC, Lee VT, Dietrich LEP. 2017. Electron-shuttling antibiotics structure bacterial communities by modulating cellular levels of c-di-GMP. Proc Natl Acad Sci USA 114:E5236–E5245. doi:10.1073/pnas.1700264114. 28607054 PMC5495239

[35] Ha D-G, Richman ME, O’Toole GA. 2014. Deletion mutant library for investigation of functional outputs of cyclic diguanylate metabolism in *Pseudomonas aeruginosa* PA14. Appl Environ Microbiol 80:3384–3393. doi:10.1128/AEM.00299-14. 24657857 PMC4018857

[36] Manzo J, Cocotl-Yanez M, Tzontecomani T, Martinez VM, Bustillos R, Velasquez C, Goiz Y, Solis Y, Lopez L, Fuentes LE, Nunez C, Segura D, Espin G, Castaneda M. 2011. Post-transcriptional regulation of the alginate biosynthetic gene algD by the Gac/Rsm system in Azotobacter vinelandii. J Mol Microbiol Biotechnol 21:147–159. doi:10.1159/000334244. 22286042

[37] Lalaouna D, Fochesato S, Sanchez L, Schmitt-Kopplin P, Haas D, Heulin T, Achouak W. 2012. Phenotypic switching in *Pseudomonas brassicacearum* involves GacS- and GacA-dependent Rsm small RNAs. Appl Environ Microbiol 78:1658–1665. doi:10.1128/AEM.06769-11. 22247157 PMC3298130

[38] Khaledi A, Weimann A, Schniederjans M, Asgari E, Kuo TH, Oliver A, Cabot G, Kola A, Gastmeier P, Hogardt M, Jonas D, Mofrad MR, Bremges A, McHardy AC, Haussler S. 2020. Predicting antimicrobial resistance in Pseudomonas aeruginosa with machine learning-enabled molecular diagnostics. EMBO Mol Med 12:e10264. doi:10.15252/emmm.201910264. 32048461 PMC7059009

[39] Hornischer K, Khaledi A, Pohl S, Schniederjans M, Pezoldt L, Casilag F, Muthukumarasamy U, Bruchmann S, Thoming J, Kordes A, Häussler S. 2019. BACTOME-a reference database to explore the sequence- and gene expression-variation landscape of *Pseudomonas aeruginosa* clinical isolates. Nucleic Acids Res 47:D716–D720. doi:10.1093/nar/gky895. 30272193 PMC6324029

[40] Markussen T, Marvig RL, Gómez-Lozano M, Aanæs K, Burleigh AE, Høiby N, Johansen HK, Molin S, Jelsbak L. 2014. Environmental heterogeneity drives within-host diversification and evolution of *Pseudomonas aeruginosa*. mBio 5:e01592-14. doi:10.1128/mBio.01592-14. 25227464 PMC4172072

[41] Ashish A, Paterson S, Mowat E, Fothergill JL, Walshaw MJ, Winstanley C. 2013. Extensive diversification is a common feature of *Pseudomonas aeruginosa* populations during respiratory infections in cystic fibrosis. J Cyst Fibros 12:790–793. doi:10.1016/j.jcf.2013.04.003. 23642644 PMC3851688

[42] Oliver A, Canton R, Campo P, Baquero F, Blazquez J. 2000. High frequency of hypermutable *Pseudomonas aeruginosa* in cystic fibrosis lung infection. Science 288:1251–1254. doi:10.1126/science.288.5469.1251. 10818002

[43] D’Argenio DA, Calfee MW, Rainey PB, Pesci EC. 2002. Autolysis and autoaggregation in *Pseudomonas aeruginosa* colony morphology mutants. J Bacteriol 184:6481–6489. doi:10.1128/JB.184.23.6481-6489.2002. 12426335 PMC135425

[44] Ren B, Shen H, Lu ZJ, Liu H, Xu Y. 2014. The *phzA2-G2* transcript exhibits direct RsmA-mediated activation in *Pseudomonas aeruginosa* M18. PLoS One 9:e89653. doi:10.1371/journal.pone.0089653. 24586939 PMC3933668

[45] Goodman AL, Kulasekara B, Rietsch A, Boyd D, Smith RS, Lory S. 2004. A signaling network reciprocally regulates genes associated with acute infection and chronic persistence in Pseudomonas aeruginosa. Dev Cell 7:745–754. doi:10.1016/j.devcel.2004.08.020. 15525535

[46] Irie Y, Starkey M, Edwards AN, Wozniak DJ, Romeo T, Parsek MR. 2010. *Pseudomonas aeruginosa* biofilm matrix polysaccharide Psl is regulated transcriptionally by RpoS and post-transcriptionally by RsmA. Mol Microbiol 78:158–172. doi:10.1111/j.1365-2958.2010.07320.x. 20735777 PMC2984543

[47] Duffy BK, Defago G. 2000. Controlling instability in *gacS-gacA* regulatory genes during inoculant production of *Pseudomonas fluorescens* biocontrol strains. Appl Environ Microbiol 66:3142–3150. doi:10.1128/AEM.66.8.3142-3150.2000. 10919762 PMC92126

[48] Achouak W, Conrod S, Cohen V, Heulin T. 2004. Phenotypic variation of *Pseudomonas brassicacearum* as a plant root-colonization strategy. Mol Plant Microbe Interact 17:872–879. doi:10.1094/MPMI.2004.17.8.872. 15305608

[49] Jonas K, Edwards AN, Ahmad I, Romeo T, Römling U, Melefors O. 2010. Complex regulatory network encompassing the Csr, c-di-GMP and motility systems of *Salmonella Typhimurium*. Environ Microbiol 12:524–540. doi:10.1111/j.1462-2920.2009.02097.x. 19919539 PMC2888478

[50] Jonas K, Edwards AN, Simm R, Romeo T, Römling U, Melefors O. 2008. The RNA binding protein CsrA controls cyclic di-GMP metabolism by directly regulating the expression of GGDEF proteins. Mol Microbiol 70:236–257. doi:10.1111/j.1365-2958.2008.06411.x. 18713317 PMC2735045

[51] Lu XH, An SQ, Tang DJ, McCarthy Y, Tang JL, Dow JM, Ryan RP. 2012. RsmA regulates biofilm formation in *Xanthomonas campestris* through a regulatory network involving cyclic di-GMP and the Clp transcription factor. PLoS One 7:e52646. doi:10.1371/journal.pone.0052646. 23285129 PMC3528676

[52] Moscoso JA, Mikkelsen H, Heeb S, Williams P, Filloux A. 2011. The Pseudomonas aeruginosa sensor RetS switches type III and type VI secretion via c-di-GMP signalling. Environ Microbiol 13:3128–3138. doi:10.1111/j.1462-2920.2011.02595.x. 21955777

[53] Moscoso JA, Jaeger T, Valentini M, Hui K, Jenal U, Filloux A. 2014. The diguanylate cyclase SadC is a central player in Gac/Rsm-mediated biofilm formation in *Pseudomonas aeruginosa*. J Bacteriol 196:4081–4088. doi:10.1128/JB.01850-14. 25225264 PMC4248864

[54] Goodman AL, Merighi M, Hyodo M, Ventre I, Filloux A, Lory S. 2009. Direct interaction between sensor kinase proteins mediates acute and chronic disease phenotypes in a bacterial pathogen. Genes Dev 23:249–259. doi:10.1101/gad.1739009. 19171785 PMC2648536

[55] Merighi M, Lee VT, Hyodo M, Hayakawa Y, Lory S. 2007. The second messenger bis-(3'-5')-cyclic-GMP and its PilZ domain-containing receptor Alg44 are required for alginate biosynthesis in *Pseudomonas aeruginosa*. Mol Microbiol 65:876–895. doi:10.1111/j.1365-2958.2007.05817.x. 17645452

[56] Liang Z, Rybtke M, Kragh KN, Johnson O, Schicketanz M, Zhang YE, Andersen JB, Tolker-Nielsen T. 2022. Transcription of the alginate operon in *Pseudomonas aeruginosa* is regulated by c-di-GMP. Microbiol Spectr 10:e00675-22. doi:10.1128/spectrum.00675-22. 35862969 PMC9431422

[57] Fusco G, Minelli A. 2010. Phenotypic plasticity in development and evolution: facts and concepts. Introduction. Philos Trans R Soc Lond B Biol Sci 365:547–556. doi:10.1098/rstb.2009.0267. 20083631 PMC2817147

[58] Jablonka E, Oborny B, Molnar I, Kisdi E, Hofbauer J, Czaran T. 1995. The adaptive advantage of phenotypic memory in changing environments. Philos Trans R Soc Lond B Biol Sci 350:133–141. doi:10.1098/rstb.1995.0147. 8577857

[59] Kordes A, Grahl N, Koska M, Preusse M, Arce-Rodriguez A, Abraham W-R, Kaever V, Häussler S. 2019. Establishment of an induced memory response in Pseudomonas aeruginosa during infection of a eukaryotic host. ISME J 13:2018–2030. doi:10.1038/s41396-019-0412-1. 30952997 PMC6775985

[60] Rybtke MT, Borlee BR, Murakami K, Irie Y, Hentzer M, Nielsen TE, Givskov M, Parsek MR, Tolker-Nielsen T. 2012. Fluorescence-based reporter for gauging cyclic di-GMP levels in *Pseudomonas aeruginosa*. Appl Environ Microbiol 78:5060–5069. doi:10.1128/AEM.00414-12. 22582064 PMC3416407

[61] Lunter G, Goodson M. 2011. Stampy: a statistical algorithm for sensitive and fast mapping of Illumina sequence reads. Genome Res 21:936–939. doi:10.1101/gr.111120.110. 20980556 PMC3106326

[62] Love MI, Huber W, Anders S. 2014. Moderated estimation of fold change and dispersion for RNA-seq data with DESeq2. Genome Biol 15:550. doi:10.1186/s13059-014-0550-8. 25516281 PMC4302049

[63] Hulsen T, de Vlieg J, Alkema W. 2008. BioVenn - a web application for the comparison and visualization of biological lists using area-proportional Venn diagrams. BMC Genomics 9:488. doi:10.1186/1471-2164-9-488. 18925949 PMC2584113

[64] Xu H, Lin W, Xia H, Xu S, Li Y, Yao H, Bai F, Zhang X, Bai Y, Saris P, Qiao M. 2005. Influence of ptsP gene on pyocyanin production in Pseudomonas aeruginosa. FEMS Microbiol Lett 253:103–109. doi:10.1016/j.femsle.2005.09.027. 16239083

[65] Spangler C, Bohm A, Jenal U, Seifert R, Kaever V. 2010. A liquid chromatography-coupled tandem mass spectrometry method for quantitation of cyclic di-guanosine monophosphate. J Microbiol Methods 81:226–231. doi:10.1016/j.mimet.2010.03.020. 20385176

[66] Blanka A, Düvel J, Dötsch A, Klinkert B, Abraham WR, Kaever V, Ritter C, Narberhaus F, Häussler S. 2015. Constitutive production of c-di-GMP is associated with mutations in a variant of Pseudomonas aeruginosa with altered membrane composition. Sci Signal 8:ra36. doi:10.1126/scisignal.2005943. 25872871

[67] Müsken M, Di Fiore S, Dötsch A, Fischer R, Häussler S. 2010. Genetic determinants of Pseudomonas aeruginosa biofilm establishment. Microbiology (Reading) 156:431–441. doi:10.1099/mic.0.033290-0. 19850623

[68] Schinner S, Preusse M, Kesthely C, Häussler S. 2021. Analysis of the organization and expression patterns of the convergent Pseudomonas aeruginosa lasR/rsaL gene pair uncovers mutual influence. Mol Microbiol 115:643–657. doi:10.1111/mmi.14628. 33073409

[69] Martin M. 2011. Cutadapt removes adapter sequences from high-throughput sequencing reads. EMBnet J 17:10–12. doi:10.14806/ej.17.1.200.

[70] Langmead B, Salzberg SL. 2012. Fast gapped-read alignment with Bowtie 2. Nat Methods 9:357–359. doi:10.1038/nmeth.1923. 22388286 PMC3322381

[71] Liao Y, Smyth GK, Shi W. 2014. featureCounts: an efficient general purpose program for assigning sequence reads to genomic features. Bioinformatics 30:923–930. doi:10.1093/bioinformatics/btt656. 24227677

[72] Robinson MD, McCarthy DJ, Smyth GK. 2010. edgeR: a Bioconductor package for differential expression analysis of digital gene expression data. Bioinformatics 26:139–140. doi:10.1093/bioinformatics/btp616. 19910308 PMC2796818

[73] Kumar L, Futschik ME. 2007. Mfuzz: a software package for soft clustering of microarray data. Bioinformation 2:5–7. doi:10.6026/97320630002005. 18084642 PMC2139991

