# Supplementary material for: Distinct Long- and Short-Term Adaptive Mechanisms in Pseudomonas aeruginosa
Source: Microbiol Spectr. 2022 Nov 14;10(6):e03043-22. doi: 10.1128/spectrum.03043-22 (PMC9769816; doi:10.1128/spectrum.03043-22)
Supplement: Supplemental file 4 — Supplemental material. Download spectrum.03043-22-s0004.pdf, PDF file, 0.7 MB [file spectrum.03043-22-s0004.pdf]

Supplemental information

Table S1: Mutations in *gacA* and *gacS* found in overall 20 independently generated revertants of the SCV24

REV	GENE	POS N	POS AA	Nucleotide found in		Amino acid in		Variation
				SCV24	REV	SCV24	REV	
REVn	*gacA*	281	94	GCAGGCCGGC GCCGCCGGCT ACATGACCAA GGGCGCGGG GCTGGAGGAA ATGGTCCAGG CTATTCGCCA GGTCTTCGCC GGCCAGCGCT ATAT	---	Frameshift		Deletion
**REVa***	***gacA***	**391**	**131**	**C**	**T**	**Q**	**STOP**	**SNP**
REVp	*gacA*	430	141	C	T	Q	STOP	SNP
REVg	*gacA*	538	180	C	T	T	I	SNP
REVs	*gacA*	632	208	GC	GGC	Frameshift		Insertion
REVd	*gacS*	379	127	C	T	L	L	SNP
**REVc***	***gacS***	**629**	**210**	**G**	**GAAGG**	**Frameshift**		**Insertion**
REVd	*gacS*	879	293	C	A	H	Q	SNP
REVf	*gacS*	1090	364	C	T	Q	STOP	SNP
REVk	*gacS*	1120	374	G	A	A	T	SNP
REVq	*gacS*	1337	446	G	A	G	D	SNP
REVh	*gacS*	1420	474	G	A	G	S	SNP
REVv	*gacS*	1426	476	G	A	G	S	SNP
REVm	*gacS*	1549	517	G	T	G	STOP	SNP
REVt	*gacS*	1620	541	CT	CCT	Frameshift		Insertion
**REVb***	***gacS***	**1787**	**596**	**C**	**A**	**S**	**STOP**	**SNP**
REVi	*gacS*	1838	613	CACCGAGCAG GCGCAATACC ACGCGACCCT GCCCGAC	CACCGAGCAG GCGCAATACC ACGCGACCCT GCCCGACCAC CGAGCAGGCG CAATACCACG CGACCCTGCC CGAC	Frameshift		Duplication
REVe, REVs	*gacS*	2477	826	A	C	D	A	SNP
REVe, REVs	*gacS*	2484	827	TGCTG	---	Frameshift		Deletion
REVj	---	---	---	---	---	---	---	---
REVo	---	---	---	---	---	---	---	---
REVr	---	---	---	---	---	---	---	---
REVu	---	---	---	---	---	---	---	---

POS = position, N = nucleotide, AA = amino acid, REVa,b,c, which have been characterized in more detail in this study, are in bold

Table S2: Strains and plasmids used in this study

Strain	Description	Reference
Escherichia coli		
DH5α	F^- *endA1*, *glnV44*, *thi-1*, *recA1*, *relA1*, *gyrA*96, *deoR*, *nupG*, Φ80*dlacZ*ΔM15, Δ(*lacZYA*-*argF*)U169, *hsdR17*($r_K^- m_K^+$), λ–	[1]
Pseudomonas aeruginosa		
SCV24	Clinical isolate	This study
REVa-v	*In vitro* generated from SCV24	This study
PA14	Wild type	[2]
**Plasmid**		
pUCP20T	Shuttle vector, Amp^R	[3]
pUCP20T::*gacA*	PA14 *gacA* gene cloned into MCS of pUCP20T via *Eco*RI/*Xba*I	This study
pUCP20T::*gacS*	PA14 *gacS* gene cloned into MCS of pUCP20T via *Xba*I/*Hind*III	This study
pUCP20T::*gacAgacS*	PA14 *gacS* gene cloned into MCS of pUCP20T::*gacA* via *Xba*I/*Hind*III	This study
pCdrA-*gfp*(ASV)^C	PcdrA-RBSII-*gfp*(ASV)-T0-T1, Cm^R, Amp^R	[4]

Amp^R, ampicillin resistant; Cm^R, chloramphenicol resistant

Table S3: Primers used in this study

Primer Name	Primer Sequence
**PCR Primer - Cloning**	
*gacA*_EcoRI_fw	5' – CCGGAATTCGTGATTAAGGTGCTGGTGGTCG
*gacA*_XbaI_rv	5' – GCTCTAGACTAGCTGGCGGCATCGAC
*gacS*_XbaI_fw	5' – GCTCTAGAGTGTTCAAGGATCTCGGCATCA
*gacS*_HindIII_rv	5' – CCCAAGCTTTCAGAGTTCGCTGGAGTCGAG
**Sequencing control – Pyrosequencing**	
*gacS*_Seq P. Fw1	5' – ATCAACCGCATGGCGGAAAC
*gacS*_Seq P. Fw2	5' – TGGATCAGCCTGAGTCTGCC
*gacS*_Seq P. Fw3	5' – ATCGCGCTCACCGCACAT
*gacS*_Seq P. Rv1	5' – TGAAGACCAGGTCGAAGCGC
*gacS*_Seq P. Rv2	5' – TTGCCGCGACAGTGAGTTGT

Engineered restriction sites are underlined

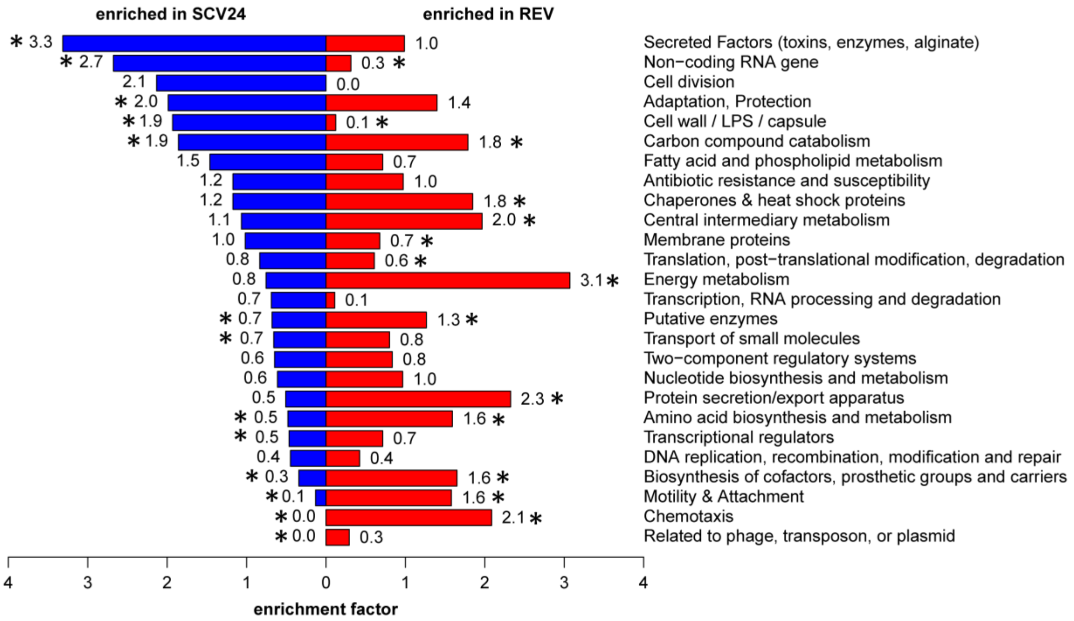

Figure S1: Transcriptional analysis uncovers phenotypic switching of revertants.

Genes that were differentially expressed under rich medium conditions (log2 fold change ≥ 1.5) between the evolved revertants (REVa and REVb were treated as replicates) and the ancestor SCV24 were assigned to PseudoCAP categories (a complete list of the differentially expressed genes can be found in Supplementary Table S4). The ratio of the abundance of differentially regulated genes (blue, enriched in SCV24; red, enriched in REV) within the distinct PseudoCAP categories to the abundance of genes in the respective category was determined, as well as the ratio of the abundance of genes within the respective category to the total number of expressed genes. The enrichment factor depicted here represents the ratio of these two values. Asterisks indicate parameters that show statistically significant differences ($p < 0.05$) as determined by hypergeometrical distribution.

Table S4: Overlap of genes that were found to be differentially regulated in the SCV24 as compared to its revertants REVa and REVb. 405 genes with a pval ≤ 0.05 and log_2 FC≥1.5; log_2 FC≤ -1.5 are listed.

SCV24 vs REV						
PA14_18580	PA14_40660	PA14_31720	PA14_10530	PA14_70900	PA14_13190	PA14_54080
PA14_18510	PA14_36540	PA14_10540	PA14_31390	PA14_39890	PA14_32670	PA14_18070
PA14_18520	PA14_00860	PA14_41420	PA14_19320	PA14_00580	PA14_67250	PA14_42310
PA14_18550	PA14_00890	PA14_69620	PA14_33610	PA14_13430	PA14_12680	PA14_61870
PA14_18480	PA14_00940	PA14_57060	PA14_23520	PA14_50570	PA14_00300	PA14_51220
PA14_28520	PA14_00910	PA14_15770	PA14_39970	PA14_09480	PA14_50880	PA14_06770
PA14_01080	PA14_01140	PA14_00490	PA14_01910	PA14_31700	PA14_19870	PA14_37060
PA14_00740	PA14_36550	PA14_21450	PA14_36980	PA14_26165	PA14_72500	PA14_70330
PA14_18430	PA14_32240	PA14_54830	PA14_64750	PA1530.1	PA14_06890	PA14_20560
PA14_18500	PA14_00875	PA14_04180	PA14_19030	PA14_61520	PA14_51170	PA14_42340
PA14_31430	PA14_01200	PA14_29400	PA14_29820	PA14_09420	PA14_00290	PA14_70690
PA14_18450	PA14_72900	PA14_33690	PA14_20970	PA14_54790	PA14_61950	PA14_02630
PA14_20060	PA14_23130	PA14_28120	PA14_03000	PA14_23100	PA14_56640	PA14_67840
PA14_18470	PA14_01170	PA14_33280	PA14_62780	PA14_08580	PA14_51390	PA14_42290
PA14_18565	PA14_00925	PA14_21830	PA14_27850	PA14_08590	PA14_13720	PA14_37000
PA14_01060	PA14_07330	PA14_02830	PA14_04040	PA14_00570	PA14_52120	PA14_06700
PA14_02760	PA14_01190	PA14_20940	PA14_33810	PA14_28040	PA14_13170	PA14_42440
PA14_18380	PA3621.1	PA14_33820	PA14_35070	PA14_02910	PA14_54690	PA14_28410
PA14_01070	PA14_21680	PA14_20920	PA14_54820	PA14_00510	PA14_45010	PA14_39060
PA14_00990	PA14_03050	PA14_20900	PA14_16190	PA14_20070	PA14_53970	PA14_06790
PA14_18410	PA14_29420	PA14_49290	PA14_52290	PA14_62080	PA14_18810	PA14_29640
PA14_10220	PA14_00820	PA14_10790	PA14_63420	PA14_46370	PA14_01730	PA14_06690
PA14_01110	PA14_36740	PA14_03610	PA14_46080	PA14_39880	PA14_12450	PA14_42620
PA14_01020	PA14_36570	PA14_66540	PA14_43910	PA14_58240	PA14_11740	PA14_29650
PA14_01100	PA14_32080	PA14_00970	PA14_64740	PA14_54260	PA14_37140	PA14_42570
PA14_02900	PA14_00830	PA14_35590	PA14_33700	PA14_62070	PA14_49690	PA14_42450
PA14_33480	PA14_31820	PA14_72760	PA14_10560	PA14_07110	PA14_13830	PA14_16990
PA14_14660	PA14_00960	PA14_02850	PA14_21000	PA14_58290	PA14_20590	PA14_64050
PA14_01030	PA14_28010	PA14_31750	PA14_19170	PA14_37760	PA14_71080	PA14_06680
PA14_06875	PA14_29190	PA14_31740	PA14_30730	PA14_28920	PA14_22420	PA14_06740
PA14_01040	PA14_33520	PA14_02840	PA14_58260	PA14_55635	PA14_56660	PA14_20190
PA14_24440	PA14_01230	PA14_43740	PA14_30680	PA14_45910	PA14_40630	PA14_06860
PA14_01010	PA14_01160	PA14_24860	PA14_07570	PA14_54760	PA14_05860	PA14_06750
PA14_02770	PA14_29200	PA14_19990	PA14_21470	PA14_42910	PA14_20570	PA14_42320
PA14_54740	PA14_32100	PA14_45700	PA14_32130	PA14_07560	PA14_46300	PA14_64530
PA14_17000	PA14_28000	PA14_20950	PA14_01540	PA14_68610	PA14_00280	PA14_29320
PA14_36850	PA14_33260	PA14_10500	PA14_63080	PA14_61290	PA14_53950	PA14_61000
PA14_00710	PA14_33500	PA14_20610	PA14_13420	PA14_39960	PA14_35360	PA14_37040
PA14_33160	PA14_02810	PA14_17510	PA14_20980	PA14_13350	PA14_27740	PA14_06670
PA14_40080	PA4704-4705	PA14_56090	PA14_56190	PA14_49930	PA14_53470	PA14_42460
PA14_54750	PA14_32190	PA14_31760	PA14_13360	PA14_04350	PA14_41990	PA14_06650
PA14_40650	PA14_10490	PA14_19020	PA14_22000	PA14_13390	PA14_12440	PA14_06810
PA14_68460	PA14_43310	PA14_02930	PA14_26600	PA14_39910	PA14_07430	PA14_39420
PA14_32140	PA14_32060	PA14_45100	PA14_21020	PA14_55170	PA14_52380	PA14_20180
PA14_02790	PA14_33450	PA14_06390	PA14_13380	PA14_00630	PA14_58690	PA14_00620
PA14_32150	PA14_29390	PA14_19010	PA14_53210	PA14_09460	PA14_51150	PA14_61180
PA14_33460	PA14_18985	PA14_31730	PA14_72690	PA14_09410	PA14_67540	PA14_06660
PA14_32160	PA14_02980	PA14_51500	PA14_70890	PA14_44230	PA14_51950	PA14_37270
PA14_01180	PA4704.1	PA14_13370	PA14_58610	PA14_46660	PA14_29290	PA14_37250
PA14_41300	PA14_16200	PA14_48150	PA14_34870	PA14_38340	PA14_18260	PA14_37310
PA14_32230	PA14_03030	PA14_31420	PA14_32290	PA14_00310	PA14_00560	PA14_42630
PA14_68450	PA14_28210	PA14_28130	PA14_16160	PA14_70040	PA14_37010	PA14_37260
PA14_02990	PA14_32780	PA14_10550	PA14_20020	PA14_71390	PA14_64500	PA14_20150
PA14_32220	PA14_01900	PA14_20960	PA14_50590	PA14_67150	PA14_70310	PA14_37290
PA14_46280	PA14_38350	PA14_56390	PA14_09400	PA14_30260	PA14_40570	PA14_37210
PA14_00850	PA14_01150	PA14_67830	PA14_26610	PA14_61610	PA14_37030	PA14_42600
PA14_16110	PA14_28200	PA14_48170	PA14_16180	PA14_67240	PA14_13300	PA14_20170
PA14_00900	PA14_39190	PA14_21010	PA14_24940	PA14_55110	PA14_72060	

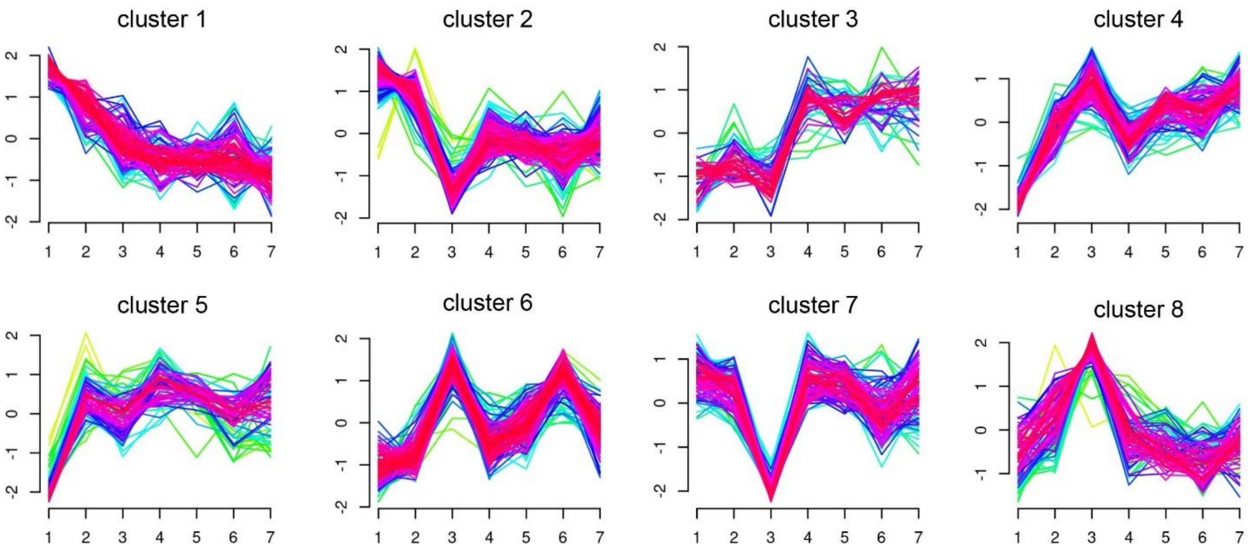

Fig S2: Cluster analysis of gene expression patterns during LB passaging of SCV24. SCV24 was passaged seven times once daily in LB medium and duplicate samples for RNA sequencing were taken at OD600 of 2 from each passage. Cluster analysis was performed using the R package Mfuzz (v2.52.0) with log2 normalized reads per gene as input. Y-axis is indicating the relative expression change and x-axis is representing the individual passages from day 1-7. Gene lists of each cluster can be found in Dataset 3.

Table S5: Phenotype distribution of 15 clinical isolates exhibiting an SCV24-like transcriptional profile. Morphology categorization was done based on colony size (ranging from very small (SCV = 0) to large (3)) and surface appearance (ranging from rough (0) over smooth (1) to clearly mucoid (2)).

Isolate	size	surface	*mucA*	*mucB*	*algU*
**MHH2419**	SCV	smooth	-	-	-
**CH4733**	SCV	mucoid	-	-	-
**MS5**	SCV	mucoid	-	Q171*	-
**MHH0147**	SCV	mucoid	-	A134V, T211A	-
**M70564993**	medium	rough	-	T211A	-
**CH3484**	large	mucoid	-	-	-
**SCV104357**	SCV	smooth	Q114*	-	-
**MHH1883**	SCV	mucoid	P76L & Q169*	A196S, T211A	-
**ESP023**	SCV	mucoid	-	-	-
**CH2678**	SCV	smooth	Q118*	-	-
**ESP011**	SCV	mucoid	Q118*	S113F	-
**CH2674**	SCV	smooth	-	T211A	-
**MS2**	SCV	smooth	-	-	-
**ESP040**	SCV	mucoid	-	T211A	-
**ESP039**	SCV	mucoid	-	T211A	-
**SCV24**	SCV	mucoid	Q117*	-	-

Isolate	size	surface	*algZ*	*algB*	*amrZ*	*algW*	*algR*	*algD*
**MHH2419**	SCV	smooth	-	-	-	-	-	-
**CH4733**	SCV	mucoid	A332T	-	-	H25R	-	-
**MS5**	SCV	mucoid	-	-	-	-	-	-
**MHH0147**	SCV	mucoid	-	-	-	D386N	-	-
**M70564993**	medium	rough	-	V76I, L382R, A393T	-	H25R, E140K, T302S, D386N	-	-

**CH3484**	large	mucoid	-	-	-	-	-	-
**SCV104357**	SCV	smooth	-	-	-	-	A122V	-
**MHH1883**	SCV	mucoid	-	L382R	-	H25R, D386N	-	-
**ESP023**	SCV	mucoid	-	-	-	-	-	-
**CH2678**	SCV	smooth	-	-	-	Q344*	-	-
**ESP011**	SCV	mucoid	-	-	-	H25R	-	-
**CH2674**	SCV	smooth	-	L382R	-	H25R, D386N	-	-
**MS2**	SCV	smooth	-	-	-	-	-	-
**ESP040**	SCV	mucoid	A133S	-	-	-	-	-
**ESP039**	SCV	mucoid	A133S	-	-	-	-	-
**SCV24**	SCV	mucoid	-	-	-	-	-	T61I

**Isolate**	**size**	**surface**	***retS***	***gacS***	***gacA***
**MHH2419**	SCV	smooth	-	-	-
**CH4733**	SCV	mucoid	-	-	-
**MS5**	SCV	mucoid	-	-	-
**MHH0147**	SCV	mucoid	-	-	-
**M70564993**	medium	rough	V46A, I324V	D361N	-
**CH3484**	large	mucoid	-	-	-
**SCV104357**	SCV	smooth	-	-	L21P
**MHH1883**	SCV	mucoid	D29A, V46A, I324V	-	-
**ESP023**	SCV	mucoid	-	-	-
**CH2678**	SCV	smooth	-	-	-
**ESP011**	SCV	mucoid	-	-	-
**CH2674**	SCV	smooth	-	D560N, D907E	-
**MS2**	SCV	smooth	-	-	-
**ESP040**	SCV	mucoid	V100A	S792T	-
**ESP039**	SCV	mucoid	V100A	S792T	-
**SCV24**	SCV	mucoid	-	-	-

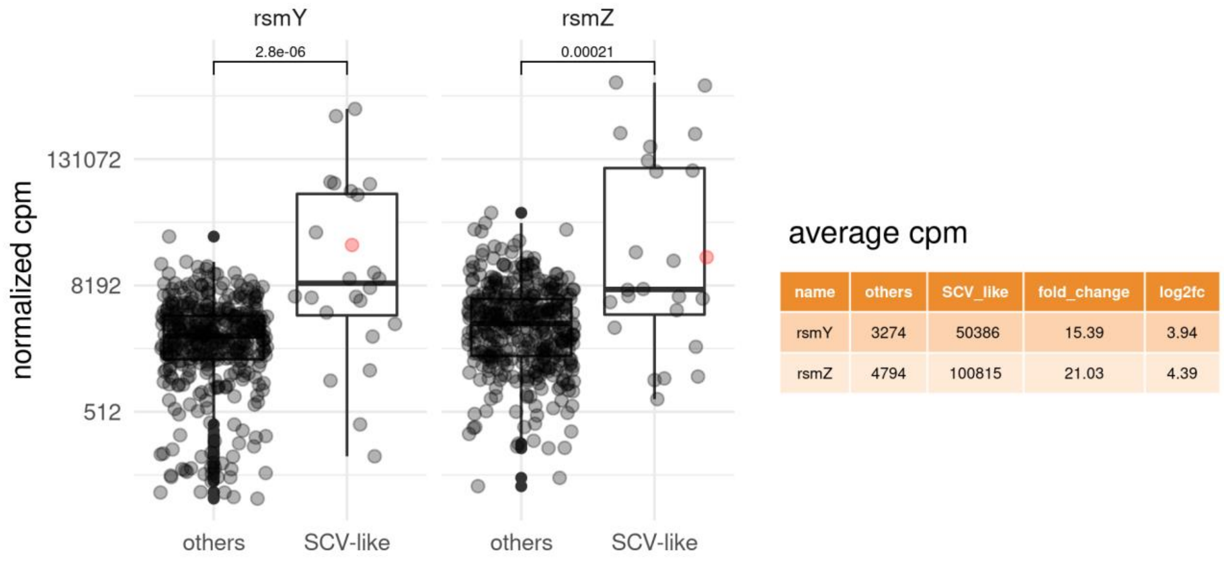

Figure S3: mRNA expression of rsmY and Z in SCV like isolates in comparison to non SCV like isolates. Normalizes counts per million (cpm) are shown. Fold change is calculates by the average cpm. Significance is calculated by unpaired ttest.the red dot represents the SCV24.

1. Woodcock DM, Crowther PJ, Doherty J, Jefferson S, DeCruz E, Noyer-Weidner M, et al. Quantitative evaluation of *Escherichia coli* host strains for tolerance to cytosine methylation in plasmid and

phage recombinants. Nucleic Acids Res. 1989;17(9):3469-78. PubMed PMID: 2657660; PubMed Central PMCID: PMCPMC317789.

2. Liberati NT, Urbach JM, Miyata S, Lee DG, Drenkard E, Wu G, et al. An ordered, nonredundant library of *Pseudomonas aeruginosa* strain PA14 transposon insertion mutants. Proc Natl Acad Sci U S A. 2006;103(8):2833-8. doi: 10.1073/pnas.0511100103. PubMed PMID: 16477005; PubMed Central PMCID: PMCPMC1413827.

3. West SE, Schweizer HP, Dall C, Sample AK, Runyen-Janecky LJ. Construction of improved *Escherichia-Pseudomonas* shuttle vectors derived from pUC18/19 and sequence of the region required for their replication in *Pseudomonas aeruginosa*. Gene. 1994;148(1):81-6. PubMed PMID: 7926843.

4. Rybtke MT, Borlee BR, Murakami K, Irie Y, Hentzer M, Nielsen TE, et al. Fluorescence-based reporter for gauging cyclic di-GMP levels in *Pseudomonas aeruginosa*. Appl Environ Microbiol. 2012;78(15):5060-9. doi: 10.1128/AEM.00414-12. PubMed PMID: 22582064; PubMed Central PMCID: PMCPMC3416407.
